# Akt enhances the vulnerability of cancer cells to VCP/p97 inhibition-mediated paraptosis

**DOI:** 10.1038/s41419-024-06434-x

**Published:** 2024-01-13

**Authors:** Dong Min Lee, In Young Kim, Hong Jae Lee, Min Ji Seo, Mi-Young Cho, Hae In Lee, Gyesoon Yoon, Jae-Hoon Ji, Seok Soon Park, Seong-Yun Jeong, Eun Kyung Choi, Yong Hyeon Choi, Chae-Ok Yun, Mirae Yeo, Eunhee Kim, Kyeong Sook Choi

**Affiliations:** 1 Department of Biochemistry and Molecular Biology, Ajou University School of Medicine, Suwon, Republic of Korea; 2 Department of Biomedical Sciences, Ajou University Graduate School of Medicine, Suwon, Republic of Korea; 3 Department of Biochemistry and Structural Biology, University of Texas Health at San Antonio, San Antonio, TX USA; 4 Greehey Children’s Cancer Research Institute, University of Texas Health at San Antonio, San Antonio, TX USA; 5 Asan Institute for Life Sciences, Department of Convergence Medicine, Asan Medical Center, University of Ulsan College of Medicine, Seoul, Korea; 6 Department of Bioengineering, College of Engineering, Hanyang University, Seoul, Korea; 7 Department of Biological Sciences, Ulsan National Institute Science and Technology, Ulsan, South Korea

**Keywords:** Cell death, Targeted therapies

## Abstract

Valosin-containing protein (VCP)/p97, an AAA+ ATPase critical for maintaining proteostasis, emerges as a promising target for cancer therapy. This study reveals that targeting VCP selectively eliminates breast cancer cells while sparing non-transformed cells by inducing paraptosis, a non-apoptotic cell death mechanism characterized by endoplasmic reticulum and mitochondria dilation. Intriguingly, oncogenic HRas sensitizes non-transformed cells to VCP inhibition-mediated paraptosis. The susceptibility of cancer cells to VCP inhibition is attributed to the non-attenuation and recovery of protein synthesis under proteotoxic stress. Mechanistically, mTORC2/Akt activation and eIF3d-dependent translation contribute to translational rebound and amplification of proteotoxic stress. Furthermore, the ATF4/DDIT4 axis augments VCP inhibition-mediated paraptosis by activating Akt. Given that hyperactive Akt counteracts chemotherapeutic-induced apoptosis, VCP inhibition presents a promising therapeutic avenue to exploit Akt-associated vulnerabilities in cancer cells by triggering paraptosis while safeguarding normal cells.

## Introduction

Cancer cells face heightened proteotoxic stress compared to their normal counterparts due to various factors, including the production of mutated proteins, the upregulation of multiprotein complex components induced by aneuploidy [1], and increased protein synthesis driven by oncogenic activation [2]. As a result, the survival of cancer cells relies heavily on the intricate machinery responsible for alleviating proteotoxic stress and maintaining proteostasis. This machinery encompasses coordinated processes like protein synthesis, folding, processing, and degradation [2, 3]. One pivotal player in these proteostasis-associated processes is Valosin-containing protein (VCP/p97), a hexameric AAA+ ATPase [4]. VCP plays a significant role in diverse cellular functions, including endoplasmic reticulum (ER)-associated degradation (ERAD) [5], mitochondrial-associated degradation (MAD) [6], and the ubiquitin-proteasome system (UPS) [7]. Notably, VCP is frequently overexpressed in various cancer types and holds promise as both a cancer prognostic biomarker and therapeutic target [8, 9]. However, the precise mechanisms by which VCP inhibition selectively eradicates cancer cells while sparing non-cancerous cells have remained elusive.

In this study, we present evidence that VCP inhibition preferentially induces cytotoxicity in breast cancer cells when compared to non-transformed cells, primarily through the induction of paraptosis. Paraptosis is a non-apoptotic cell death mechanism characterized by cytoplasmic vacuolation originating from the ER and/or mitochondria [10]. Since apoptotic pathways are often compromised in drug-resistant cancer cells, leading to therapeutic failures [11], it becomes imperative to explore strategies that promote alternative cell death mechanisms, such as paraptosis, especially in tumors that have progressed despite conventional apoptosis-targeted therapies. A comprehensive understanding of the mechanistic details of cancer cell death is crucial for devising effective therapeutic strategies. Importantly, paraptosis differs from apoptosis in that it does not involve the release of mitochondrial cytochrome c or caspase activation. While various factors, including proteasome inhibition [12–14] and thiol proteostasis impairment [15, 16], and Ca^2+^ imbalance [17], have all been implicated in paraptosis, the detailed molecular basis remains to be fully elucidated.

Our study has illuminated the pivotal role of VCP as a molecular target for inducing paraptosis in cancer cells. Mechanistically, VCP inhibition in breast cancer cells intensifies proteotoxic stress by restoring translation, thereby contributing to the occurrence of paraptosis. This process involves the activating transcription factor 4 (ATF4)/DNA damage-inducible transcript 4 (DDIT4) axis and the mechanistic target of rapamycin complex 2 (mTORC2)/Akt signaling pathways, which play a significant role in translational recovery and subsequent amplification of proteotoxic stress. Furthermore, we emphasize the critical role of eukaryotic translation initiation factor 3 subunit D (eIF3d) as a mediator for translational recovery in cancer cells exposed to proteotoxic stress. In contrast, when VCP is inhibited in non-transformed cells, it triggers translational suppression, ultimately alleviating proteotoxic stress and promoting cell survival. Considering the crucial role of hyperactive Akt, driven by oncogenes, in cancer cell survival and resistance to therapy [18], identifying vulnerabilities within specific subsets of cancer cells can pave the way for tailored therapies targeting oncogene-addicted cancer cells.

In summary, our work suggests that inducing paraptosis through VCP inhibition may open up novel therapeutic avenues for cancer cells characterized by hyperactive Akt.

## Results

### VCP is a molecular target of paraptosis

While various natural products and chemicals have been shown to induce paraptosis [19, 20], the molecular mechanisms underlying this process remain unclear. To identify potential molecular targets for inducing paraptosis, we utilized the Connectivity Map (CMap, http://cleu.io/cmap) [21], a database that links pharmacological drugs and genomic data. The CMap dataset includes transcriptome information for 17 paraptosis-inducing chemicals, such as withaferin A [22], pyrrolidine dithiocarbamate (PDTC) [23], 15-deoxy-Δ^12,14^-prostaglandin J_2_ (15d-PGJ_2_) [24], and xanthohumol [25]. We sought genetic perturbations that elicited transcriptional alterations similar to those induced by these paraptosis-inducing chemicals. VCP knockdown emerged as the top-ranked perturbagen, with actions resembling those of the examined paraptosis inducers (Supplementary Fig. 1).

**Fig. 1:**
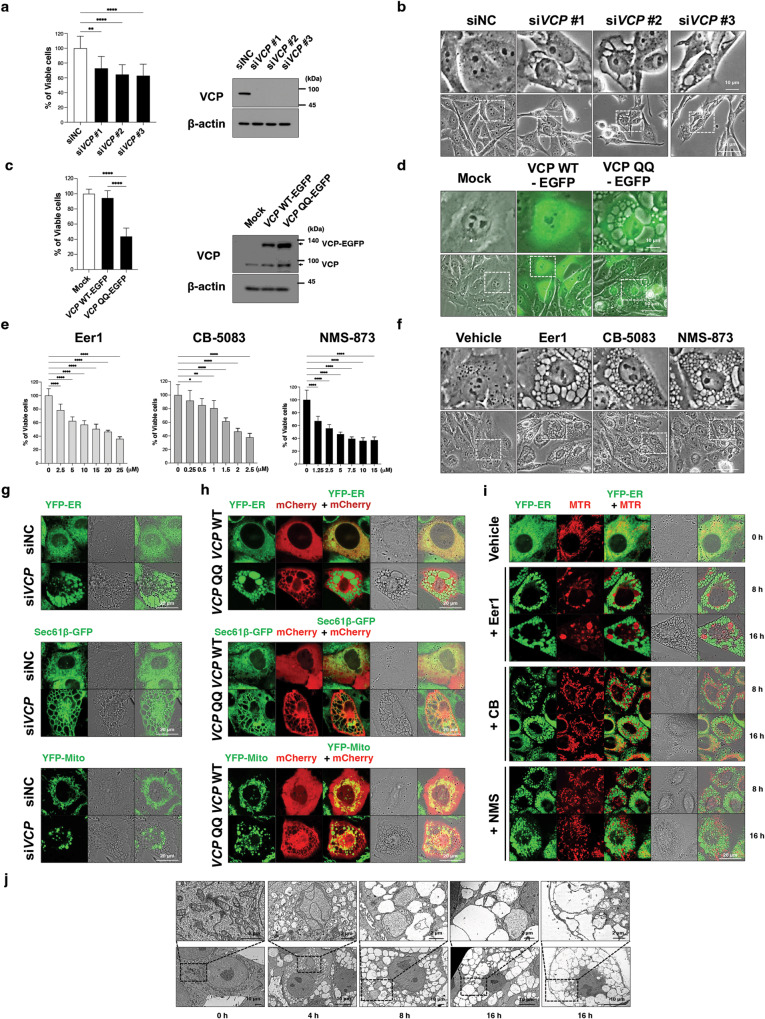
VCP impairment induces paraptotic cell death in MDA-MB 435 S cells. **a**, **b** MDA-MB 435 S cells were transfected with either a negative control siRNA (siNC) or a VCP-targeting siRNA (siVCP) and incubated with fresh medium for 48 h. **c**, **d** MDA-MB 435 S cells infected with adenovirus encoding VCP WT-EGFP or VCP QQ-EGFP for 72 h. **e**, **f** MDA-MB 435 S cells were treated with the indicated concentrations of VCP inhibitors for 24 h **e** or with 10 μM Eer1, 2 μM CB-5083, or 5 μM NMS-873 for 12 h **f**. **g**, **h** YFP-ER, Sec61β-GFP, and YFP-Mito cells were transfected with siNC or siVCP for 48 h **g** or with VCP WT-mCherry or VCP QQ-mCherry for 36 h **h**. **i** YFP-ER cells treated with 10 μM Eer1, 2 μM CB-5083, and 5 μM NMS were stained with MTR. **a** (left), **c** (left), **e** Cell viability was assessed using an IncuCyte system, as described in the Materials and Methods. The percentage of live cells was normalized to that of untreated cells (100%). Cell viability data are presented as the means ± SD of three independent experiments. *n* = 10. The *p*-values in panels **a**, **c**, and **e** were calculated by one-way ANOVA. *p < 0.05, **p < 0.01, **p < 0.001, ****p < 0.0001. ns, not significant. **a** (right), **c** (right) Western blotting of VCP using β-actin as a loading control. **b**, **f** Representative phase-contrast microscopic images. **d** Phase-contrast/fluorescence microscopy. **g**–**i** Confocal microscopy. **j** Electron microscopy of cells treated with 10 μM Eer1.

Given VCP’s crucial role in proteostasis [4–7] and the established connection between proteostatic disruption and paraptosis [13, 16, 19, 26–28], our investigation aimed to determine whether VCP knockdown alone could trigger paraptosis. We observed that VCP knockdown, executed using three independent siRNAs, led to cell death accompanied by extensive vacuolation in MDA-MB 435 S cells (Fig. 1a, b). Similar results were obtained through the adenovirus-mediated expression of a dominant-negative VCP mutant (VCP QQ; VCP^E305Q, E578Q^) [29] fused to an enhanced green fluorescent protein (EGFP) (Fig. 1c, d) or treatments with various VCP inhibitors, including eeyarestatin-1 (Eer1, an ER membrane-binding domain-containing VCP inhibitor) [30], CB-5083 (an inhibitor of the D2 ATPase domain of VCP) [31], and NMS-873 (an allosteric VCP inhibitor) [32] (Fig. 1e, f). Next, we explored whether VCP inhibition induces vacuolation originating from the ER and/or mitochondria, a hallmark of paraptosis. To visualize these organelles, we utilized YFP-ER [33], Sec61β-GFP [16], and YFP-Mito cells [33], which exhibit fluorescence in the ER lumen, ER membrane, and mitochondrial matrix, respectively, along with MitoTracker-Red (MTR) staining. We found that VCP siRNAs (Fig. 1g), mCherry-fused VCP QQ mutant (Fig. 1h), and three VCP inhibitors (Fig. 1i) commonly induced significant dilations of the ER and mitochondria. In particular, Eer1 induced the most dramatic dilation among the tested VCP inhibitors. Electron microscopy further revealed megamitochondria (giant mitochondria) and ER-derived vacuoles in Eer1-treated cells (Fig. 1j). Time-lapse imaging in Eer1-treated YFP-ER and YFP-Mito cells confirmed ER or mitochondria swelling and fusion (Supplementary Fig. 2).

**Fig. 2:**
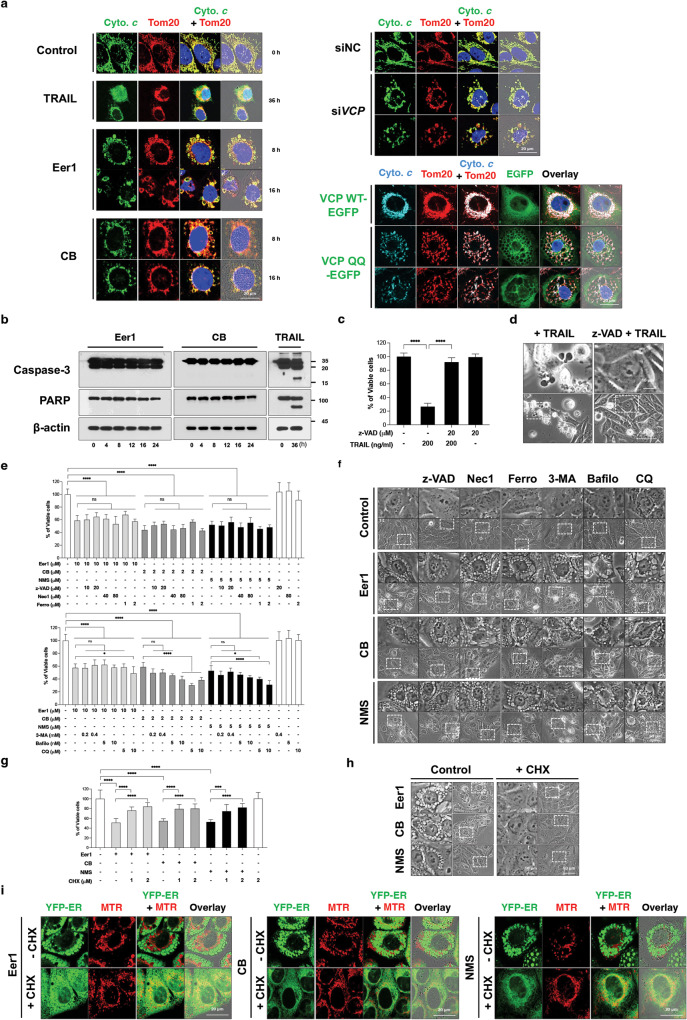
Apoptosis, necroptosis, ferroptosis, or autophagy may not critically contribute to the anticancer effect of VCP inhibition. **a** Immunocytochemistry of cytochrome *c* (cyto. *c*) and Tom20 in MDA-MB 435 S cells treated with 200 ng/ml TRAIL, 10 μM Eer1, or 2 μM CB-5083 for the indicated time durations, transfected with siNC or siVCP for 48 h, or infected with adenoviruses encoding VCP WT-EGF or VCP QQ-EGF for 48 h. **b** Western blotting of Caspase-3 and PARP in MDA-MB 435 S cells treated with 10 μM Eer1, 2 μM CB-5083, or 200 ng/μl TRAIL using β-actin as a loading control. The representative blots of two independent experiments are shown. **c**, **d** MDA-MB 435 S cells pretreated with 20 μM z-VAD-fmk were further treated with Eer1 or TRAIL for 24 h. **c** Cellular viability assay. **d** Phase-contrast microscopy. **e, f** MDA-MB 435 S cells pretreated with the indicated death inhibitors were further treated with 10 μM Eer1, 2 μM CB-5083, or 5 μM NMS for 24 h **e** or 12 h **f**. **e** Cell viability assay. **f** Representative phase-contrast microscopic images. **g**, **h** MDA-MB 435 S cells pretreated with CHX were further treated with 10 μM Eer1, 2 μM CB-5083, and 5 μM NMS for 24 h **g** or 12 h **h**. **g** Cell viability assay. **h** Phase-contrast microscopy. **i** Confocal microscopy in YFP-ER cells pretreated with 1 μM CHX, further treated with Eer1, CB-5083, or NMS for 8 h, and stained with MTR. Cell viability data **b**, **e**, **g** represent the means ± SD of three independent experiments. n = 10. The *p*-value was calculated by one-way ANOVA. *p < 0.05, **p < 0.01, **p < 0.001, ****p < 0.0001. ns, not significant.

Subsequently, we investigated the involvement of apoptosis in the anticancer effect of VCP inhibition. Unlike the release of cytochrome *c* from mitochondria observed with the apoptosis-inducer tumor necrosis factor-related apoptosis-inducing ligand (TRAIL), Eer1 or CB-5083 treatment, VCP knockdown, or VCP QQ-EGFP expression resulted in cytochrome *c* accumulation within or at the periphery of dilated mitochondria (Fig. 2a). Furthermore, caspase-3 and PARP cleavage, which was induced by TRAIL, were not notably observed by Eer1 or CB-5083 treatment (Fig. 2b). While z-VAD-fmk (a pan-caspase inhibitor) effectively blocked TRAIL-induced cell death and apoptotic morphologies (Fig. 2c, d), it did not affect vacuolation-associated cell death induced by VCP inhibitors (Fig. 2e, f). Furthermore, inhibitors of necroptosis (necrostatin-1; Nec1), ferroptosis (ferrostatin-1; Ferro), and an early-phase autophagy inhibitor (3-methyladenine; 3-MA) did not attenuate the cytotoxicity of VCP inhibitors. In contrast, late-stage autophagy inhibitors (bafilomycin A; Bafilo and chloroquine; CQ) enhanced it (Fig. 2e, f). In contrast, cycloheximide (CHX), known to block paraptosis [10], effectively inhibited mitochondria- and ER-derived vacuolation and cell death induced by the VCP inhibitors (Fig. 2g–i). Together, these results suggest that VCP inhibition predominantly induces paraptosis as a cell death mechanism in cancer cells.

### VCP inhibition triggers paraptosis in various breast cancer cell lines and in vivo xenograft mouse models, sparing non-transformed cells

**Fig. 3:**
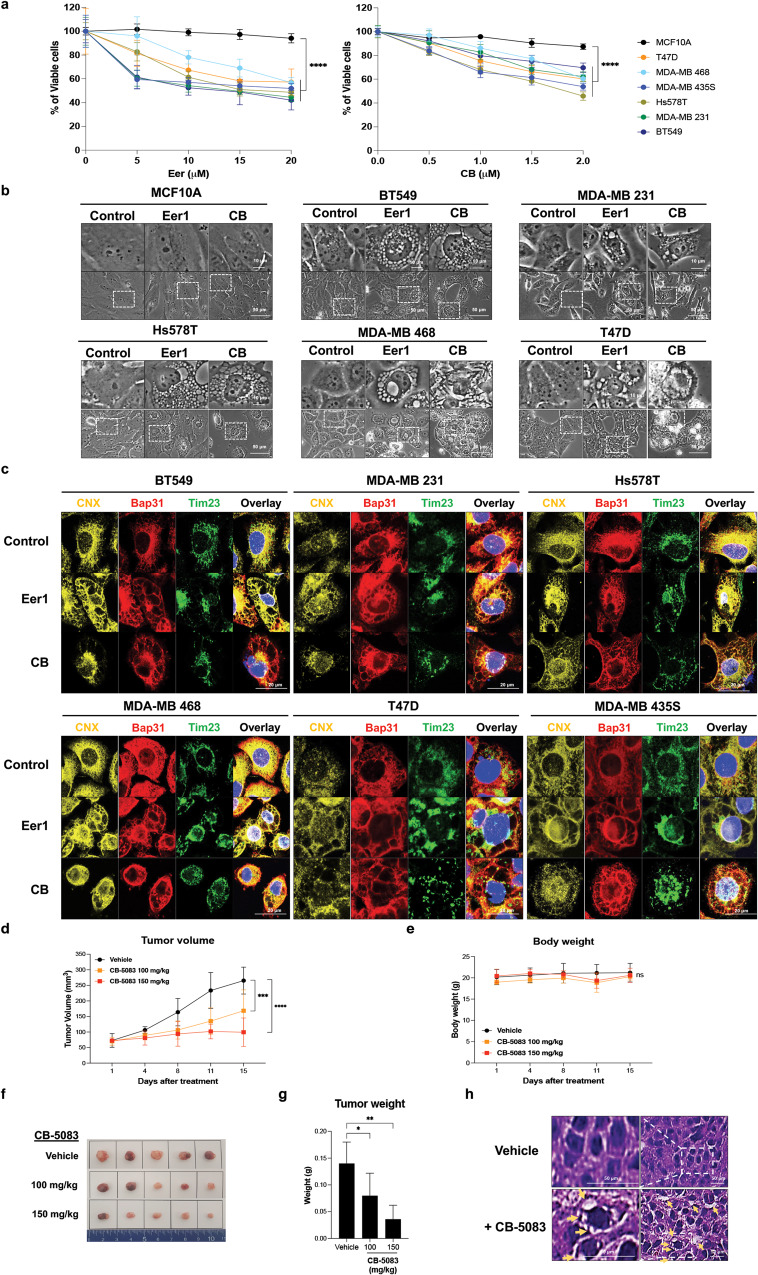
VCP inhibition is preferentially cytotoxic to cancer cells compared to non-malignant cells and induces paraptosis in vitro and in vivo. **a**–**c** Cells were treated with the indicated concentrations of Eer1 or CB-5083 for 24 h **a**, 10 μM Eer1 or 2 μM CB-5083 for 12 h **b**, **c**. **a** Cell viability assay. **b** Phase-contrast microscopy. **c** Confocal microscopy of the immunocytochemical staining of Bap31, calnexin (CNX), and Tim23. **d**–**h** Xenograft-bearing mice were treated with the indicated amounts of CB-5083 as described in the Material and Methods. The tumor volume **d** and body weight **e** were measured twice a week for 15 days, and the growth curve was plotted. On the 15^th^ day, tumors were isolated, photographed **f**, and weighed **g**. **h** H&E staining. Yellow arrows indicate the cellular vacuoles in the tumor tissues of CB-5083-treated mice. Data **a**, **d**, **e**, **g** are presented as the means ± SD of three independent experiments. The *p*-values in panels **a, d**, and **e** were calculated by two-way ANOVA, and the *p*-values in panel **g** were calculated by one-way ANOVA. *p < 0.05, **p < 0.01, ***p < 0.001, ****p < 0.0001. ns, not significant.

We further examined the impact of VCP inhibition on other breast cancer cell lines. Treatment with Eer1 or CB-5083 induced cell death accompanied by vacuolation in several breast cancer cells, including BT549, MDA-MB 231, Hs578T, MDA-MB468, and T47D cells (Fig. 3a, b). However, both Eer1 and CB-5083 displayed considerably lower cytotoxicity towards MCF10A cells, a non-tumorigenic breast epithelial cell line, without affecting their morphology (Fig. 3a, b). Immunocytochemistry of calnexin (CNX), Bap31 (an ER marker protein), and Tim23 (a mitochondrial marker protein) confirmed ER- and mitochondria-derived vacuolation in Eer1- or CB-5083-treated cancer cells (Fig. 3b, c).

To assess the in vivo effect of VCP inhibition, nude mice xenografted with MDA-MB 435 S cells were orally treated with saline or CB-5083. CB-5083 dose-dependently reduced tumor volume and weight without causing weight loss in mice (Fig. 3d–g). Hematoxylin and eosin (H&E) staining revealed severe vacuolation in the tumor tissues of CB-5083-treated mice (Fig. 3h). These results indicate that targeting VCP induces paraptosis both in vitro and in vivo and selectively affects cancer cells over non-transformed cells.

### Oncogene-driven Akt activation sensitizes non-transformed cells to VCP inhibition

**Fig. 4:**
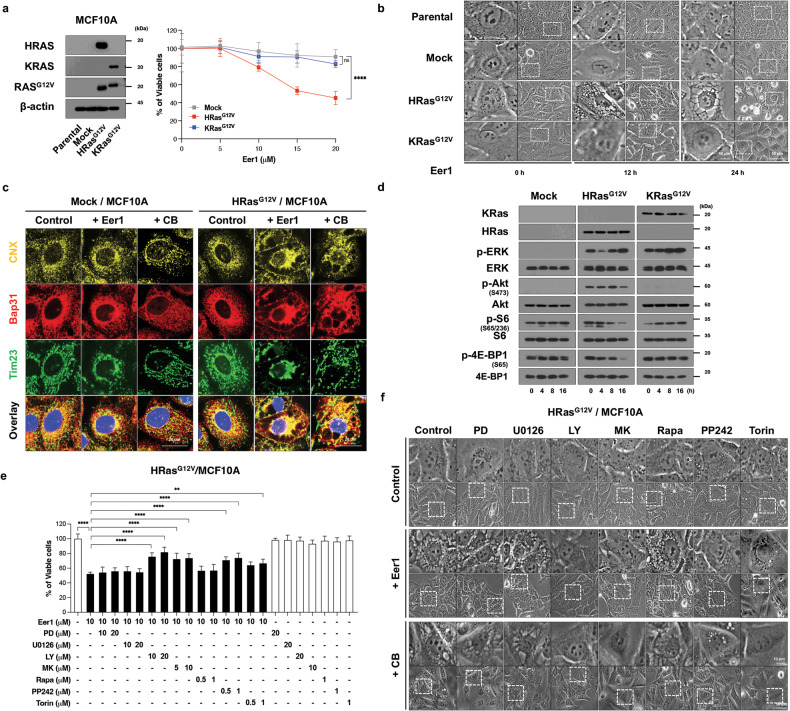
PI3K/Akt/mTOR signals may be required for the oncogenic Ras-mediated cell vulnerability to VCP inhibition. **a**–**d** Parental MCF10A cells, mock vector-transfected, HRas^G12V^-, or KRas^G12V^-expressing MCF10A cells were treated with Eer1 for 24 h **a** or for the indicated time durations **b**, **d**, or treated with 10 μM Eer1 or 2 μM CB-5083 for 12 h **c**. **e**, **f** HRas^G12V^/MCF10A cells pretreated with the indicated inhibitors were further treated with 10 μM Eer1 for 24 h **e**, or treated with 10 μM Eer1 or 2 μM CB-5083 for 12 h **f**. **a** (right), **e** Cell viability assay. **a** (left) Western blotting to confirm the overexpression of HRas^G12V^ or KRas^G12V^ using β-actin as a loading control. **b, f** Phase-contrast microscopy. **c** Immunocytochemistry of Bap31, calnexin (CNX), and Tim23. **d** Western blotting. Cell viability data **a**, **e** represent the means ± SD of three independent experiments. *n* = 10. The *p*-values in panels **a** and **e** were calculated by two-way ANOVA and one-way ANOVA, respectively. *p < 0.05, **p < 0.01, ***p < 0.001, ****p < 0.0001. ns not significant.

We explored whether the preferential sensitivity of cancer cells to VCP inhibition is linked to oncogenic activation. To investigate this, we introduced oncogenes such as KRas^G12V^ and HRas^G12V^ into non-transformed cells and examined their response to Eer1 treatment. HRas^G12V^-expressing cells (HRas^G12V^/MCF10A) showed significantly greater sensitivity to Eer1-induced cytotoxicity than KRas^G12V^-expressing cells (KRas^G12V^/MCF10A) or Mock/MCF10A cells (Fig. 4a). Interestingly, Eer1 treatment induced cell death accompanied by ER and mitochondrial dilations only in HRas^G12V^-expressing cells (Fig. 4a–c). CB-5083 treatment also induced a similar dilation of the ER and mitochondria in HRas^G12V^-expressing cells but not in Mock/MCF10A cells (Fig. 4c). Next, we investigated the downstream signaling pathways, including RAF/MEK/ERK and phosphatidylinositol-3-kinase (PI3K)/Akt/mTOR, which are associated with mutant Ras [34]. ERK activation was observed in cells expressing either HRas^G12V^ or KRas^G12V^ and further enhanced by Eer1 treatment (Fig. 4d). However, Akt activity was markedly increased only in HRas^G12V^-expressing cells and further enhanced by Eer1. Inhibition of PI3K/Akt using LY294002 (a PI3K/Akt inhibitor) or MK-2206 (an Akt inhibitor), but not inhibition of MEK (using PD98059 or U0126), blocked Eer1-induced paraptosis in HRas^G12V^/MCF10A (Fig. 4e, f). These results suggest the critical role of the PI3K/Akt pathway in sensitizing non-transformed cells to VCP inhibition. Next, we further examined the involvement of mTOR complex 1 (mTORC1) and mTOR complex 2 (mTORC2) in VCP inhibition-induced paraptosis. Inhibitors targeting both mTORC1 and mTORC2, such as PP242 and Torin1 (mTORC1/2 inhibitors), but not the mTORC1-specific inhibitor rapamycin, effectively inhibited Eer1-induced paraptosis in HRas^G12V^/MCF10A cells (Fig. 4e, f). Similar results were obtained in HRas^G12V^/MCF10A undergoing CB-5083-induced paraptosis (Fig. 4f). This was further supported by the reduction in S6 and eIF4E-binding protein 1 (4E-BP1) phosphorylation, indicative of mTORC1 activity [35], but the increase in Akt phosphorylation at S473, indicative of mTORC2 activity [35], in Eer1-treated HRas^G12V^/MCF10A cells (Fig. 4d). Collectively, these findings suggest differential roles for mTORC1 or mTORC2 in VCP inhibition-mediated paraptosis.

### Activation of mTORC2/Akt contributes to VCP inhibition-mediated paraptosis

**Fig. 5:**
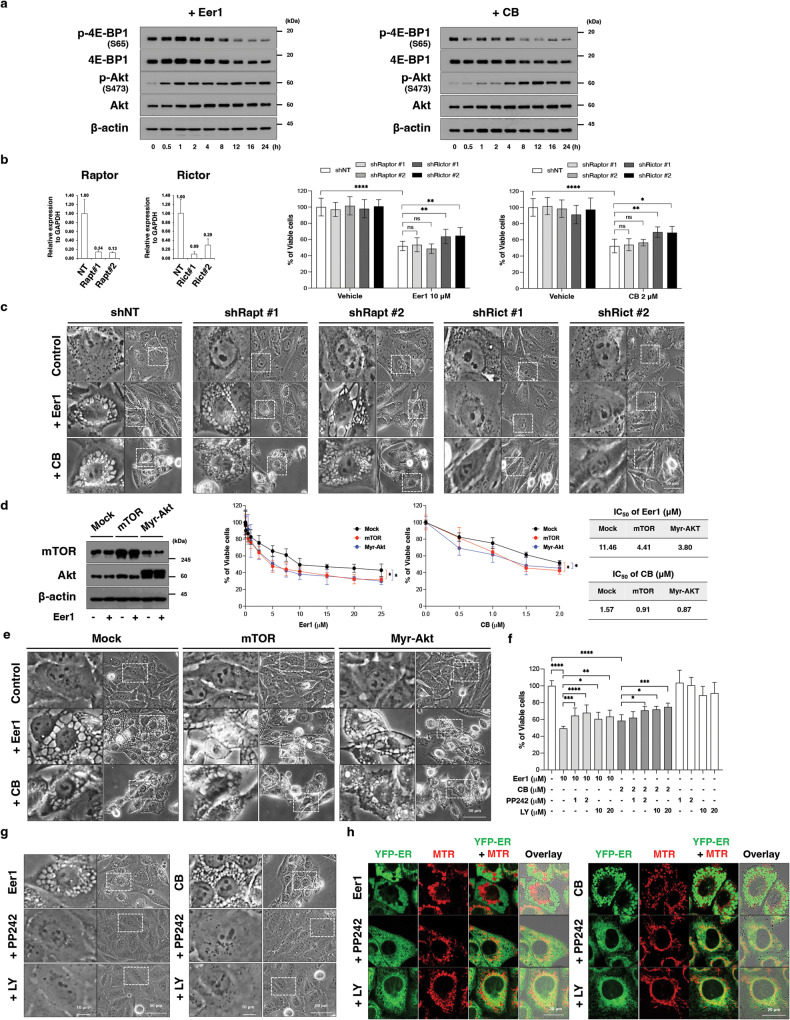
mTORC2/Akt activation is critical for VCP inhibition-induced paraptosis. **a** Western blotting of the proteins associated with mTOR signals in 10 μM Eer1- or 2 μM CB-5083-treated MDA-MB 435 S cells for the indicated time durations. Representative blots of two independent experiments are shown. **b**, **c** MDA-MB 435 S cells transduced with the lentivirus encoding shRNA against *Raptor* or *Rictor* gene were treated with 10 μM Eer1 or 2 μM CB-5083 12 h **b** (left) and **c** or for 24 h **b** (right). **b** (left) qRT-PCR using GAPDH as a reference gene. **b** (right) Cell viability assay. **c** Phase-contrast microscopy. **d**, **e** MDA-MB 435 S cells transfected with Mock vector or the plasmid encoding mTOR or Myr-Akt were treated with 10 μM Eer1 or 2 μM CB-5083 for 24 h **d** or 12 h **e**. **d** (left) Western blotting. **d** (middle) Cell viability assay. **d** (right) IC_50_ of Eer1 or CB-5083 in the respective cells was assessed. **e** Phase-contrast microscopy. **f**, **g** MDA-MB 435 S cells pretreated with the indicated inhibitors were further treated with 10 μM Eer1 or 2 μM CB-5083 for 24 h **f** or 12 h **g**. **f** Cell viability assay. **g** Phase-contrast microscopy. **h** Confocal microscopy in YFP-ER cells pretreated with 1 μM PP242 or 10 μM LY294002, further treated with 10 μM Eer1 or 2 μM CB-5083 for 8 h, and stained with MTR. Cell viability data (**b** (right), **d** (middle), **f** are presented as the means ± SD of three independent experiments. *n* = 10. The *p*-values in panel **b** (right) and **f** were calculated by one-way ANOVA. The *p*-values in panel **d** (middle) were calculated by two-way ANOVA. **p < 0.01, ****p < 0.0001. ns not significant.

We investigated whether mTOR signaling plays a similar role in cancer cells undergoing VCP inhibition-induced paraptosis. In MDA-MB 435 cells, Eer1 or CB-5083 treatment led to progressive Akt phosphorylation while reducing 4E-BP1 phosphorylation (Fig. 5a), indicating mTORC2 activation and mTORC1 inhibition. Knockdown of Rictor (a component of mTORC2) but not that of Raptor (a component of mTORC1) significantly attenuated Eer1- or CB-5083-mediated cytotoxicity and vacuolation (Fig. 5b, c). Conversely, overexpression of mTOR and constitutively active Akt (myristoylated Akt (Myr-Akt)) potentiated Eer1- or CB-5083-induced cytotoxicity and vacuolation (Fig. 5d, e). Pretreatment of cells with PP242 or LY294002 effectively inhibited Eer1- or CB-5083-induced paraptosis (Fig. 5f–h). These results suggest that hyperactive mTORC2/Akt signaling contributes significantly to VCP inhibition-mediated paraptosis.

### The ATF4/DDIT4 axis plays a crucial role in Akt activation and subsequent paraptosis upon VCP inhibition

**Fig. 6:**
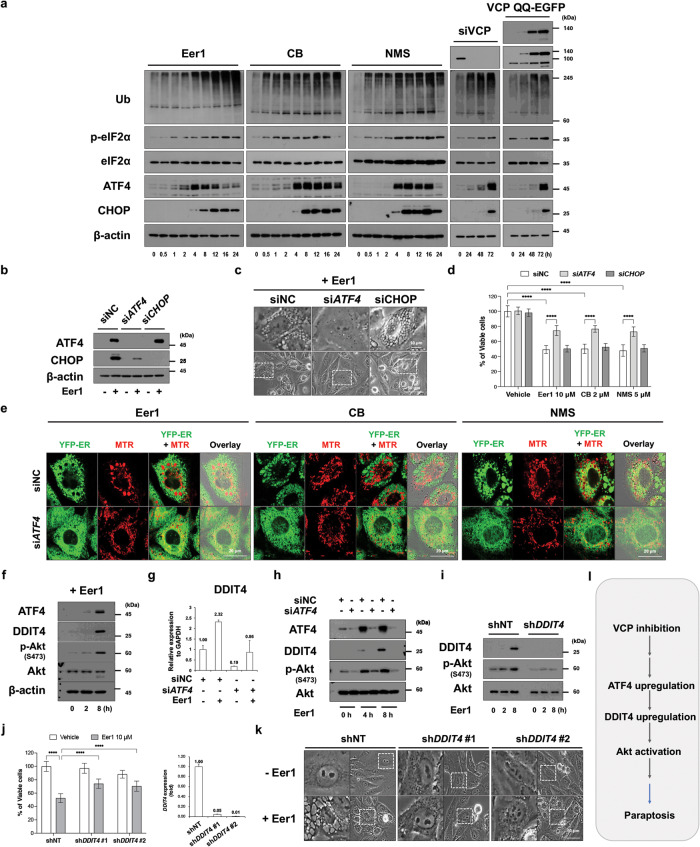
The ATF4/DDIT4 axis is crucial for VCP inhibition-mediated paraptosis, affecting Akt activation. **a** Western blotting of the ISR-associated proteins in MDA-MB 435 S cells treated with 10 μM Eer1, 2 μM CB, or 5 μM NMS-873, transfected with siVCP, or infected with VCP QQ-EGFP. **b, c** MDA-MB 435 S cells transfected with siATF4 or siCHOP were treated with 10 μM Eer1 for 24 h **b** or 12 h **c**. **b** Western blotting. The representative blots of two independent experiments are shown. **c** Phase-contrast microscopy. **d** Cell viability assay in MDA-MB 435 S cells transfected with siATF4 or siCHOP were treated with the indicated VCP inhibitors for 24 h. **e** Confocal microscopy in YFP-ER cells transfected with siNC or siATF4, treated with the VCP inhibitors for 16 h, and stained with MTR. **f** Western blotting in MDA-MB 435 S cells treated with 10 μM Eer1. **g**–**k** MDA-MB 435 S cells transfected with siNC or siATF4 **g**, **h**, or those transduced with the lentivirus encoding shNT or shDDIT4 **i**–**k** were further treated with 10 μM Eer1 for 12 h **g, j** (right), **k**, indicated time points **h**, **i**, or 24 h **j** (left). **g**, **j** (right) qRT-PCR of DDIT4 using GAPDH as a reference. **h**, **i** Western blotting. **j** (left) Cell viability assay. **k** Phase-contrast microscopy. **l** Hypothetical upstream signals for VCP inhibition-mediated paraptosis. Cell viability data **d**, **j** are presented as the means ± SD of three independent experiments. *n* = 10. The *p*-values were calculated by two-way ANOVA. ****p < 0.0001.

Proteotoxic stress caused by proteostatic disruption triggers the integrated stress response (ISR) [36], converging on the phosphorylation of eukaryotic initiation factor 2α (eIF2α). This reduces cap-dependent translation while promoting the translation of specific mRNAs, including activating transcription factor 4 (ATF4) [37]. In our study, VCP inhibitors, VCP knockdown, and VCP QQ mutant expression consistently upregulated poly-ubiquitinated proteins and the components of ISR, including phosphorylated eIF2α (p-eIF2α), ATF4, and C/EBP homologous protein (CHOP), in MDA-MB 435 S cells (Fig. 6a). Among these, ATF4 was found to be crucially associated with VCP inhibition-mediated paraptosis. ATF4 knockdown effectively inhibited paraptosis induced by Eer1, CB-5083, NMS-873 (Fig. 6b–e), VCP knockdown (Supplementary Fig. 3a–c), or VCP QQ mutant expression (Supplementary Fig. 3d–f). Knockdown of ATF4 but not CHOP effectively inhibited Eer1-induced paraptosis (Fig. 6b–e). These results underscore the role of ATF4 in VCP inhibition-mediated paraptosis. To further explore the role of ATF4 in this process, we performed the transcriptomic analysis in MDA-MB 435 S cells transfected with siNC (non-targeted siRNA) or siATF4 and in the absence or presence of Eer1 (Supplementary Fig. 4a, b). We identified genes that were responsive to Eer1 (fold change of siNC+Eer1/siNC-Eer1 > 2) and highly dependent on ATF4 (fold change of siATF4+Eer1/siNC+Eer1 < -2) (Supplementary Fig. 4c). Among these ATF4 downstream targets, we further investigated the role of DNA-damage-inducible transcript 4 (DDIT4), which is known to be associated with mTORC1 inhibition and mTORC2/Akt activation [38, 39]. Our findings revealed that Eer1 upregulated DDIT4, along with ATF4 upregulation and Akt activation (Fig. 6f). ATF4 knockdown inhibited Eer1-induced DDIT4 upregulation at the mRNA and protein levels, as well as Akt activation (Fig. 6g, h). Additionally, DDIT4 knockdown effectively inhibited Eer1-induced Akt activation (Fig. 6i). Furthermore, DDIT4 knockdown significantly blocked Eer1-induced cell death and vacuolation (Fig. 6j, k). These results suggest that the ATF4/DDIT4 axis, particularly DDIT4, may mediate Akt activation in VCP inhibition-mediated paraptosis (Fig. 6l).

### mTORC2/Akt-mediated translational recovery contributes to cancer-selective cytotoxicity of VCP inhibition

**Fig. 7:**
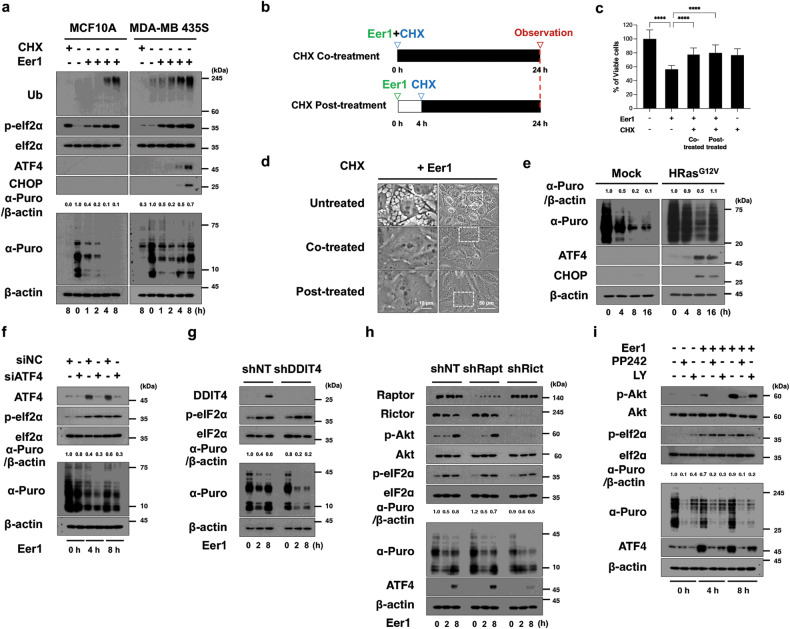
The ATF4/DDIT4 axis and mTORC2/Akt are critically involved in translational recovery during VCP inhibition-mediated paraptosis. **a** Western blotting of the ISR-associated proteins and newly synthesized puromycinylated peptides was performed in MCF10A and MDA-MB 435 S cells treated with 1 μM CHX or 10 μM Eer1. Representative blots of two independent experiments are shown. The band intensity of puromycinylated proteins was analyzed using ImageJ. **b**–**d** Eer1-treated MDA-MB 435 S cells were co-treated or post-treated (after Eer1 treatment for 4 h) with 1 μM CHX and further incubated for 24 h. **b** The treatment schedule. **c** Cell viability assay. Data are presented as the means ± SD of three independent experiments. n = 10. The *p*-value was calculated by one-way ANOVA. ****p < 0.0001. **d** Phase-contrast microscopy. **e** Mock vector-transfected or HRas^G12V^-expressing MCF10A cells were treated with 10 μM Eer1. **f**–**h** MDA-MB 435 S cells transfected with siNC or siATF4 **f** or those transduced with the lentivirus encoding the indicated shRNA **g**, **h** were further treated with Eer1. **i** MDA-MB 435 S cells pretreated with 1 μM PP242 or 10 μM LY294002 were further treated with Eer1. **e**–**i** Western blotting of the indicated proteins was performed with β-actin as a positive control. The representative blots of two independent experiments are shown.

Our investigation into the differential vulnerability of cancer and non-transformed cells to VCP inhibition revealed distinct responses in the ISR between the two cell types. While Eer1 induced robust and sustained poly-ubiquitinated protein accumulation and p-eIF2α phosphorylation in MDA-MB 435 S cells, these responses were delayed and weaker in MCF10A cells, lacking ATF4 upregulation (Fig. 7a). The protein synthesis, as assessed by the SUnSET assay [40], was strongly suppressed in Eer1-treated MCF10A cells but showed initial reduction followed by recovery in MDA-MB 435 S cells, concurrent with ATF4 upregulation (Fig. 7a). Inhibition of translation with CHX at 4 h post-Eer1 treatment effectively blocked cell death and vacuolation (Fig. 7b–d), emphasizing the importance of translational recovery. This translational recovery and ATF4/CHOP upregulation were also observed in HRas^G12V^/MCF10A cells but not in Mock/MCF10A cells (Fig. 7e), correlating with their sensitivity to VCP inhibition-induced paraptosis. These results suggest that effective translational suppression may prevent the death of non-transformed cells by alleviating VCP inhibition-mediated proteotoxic stress. However, in cancer cells with hyperactive Akt (possibly driven by oncogenic signals such as HRas^G12V^), the translational recovery under VCP inhibition-mediated proteotoxic stress may enhance proteotoxicity by increasing the accumulation of misfolded proteins in the ER and mitochondria, leading to paraptosis. Next, we investigated whether the ATF4/DDIT4 axis and mTORC2/Akt signals are linked to translational dysregulation in VCP inhibition-mediated paraptosis. Either ATF4 or DDIT4 knockdown inhibited Eer1-induced translational recovery without affecting eIF2α phosphorylation (Fig. 7f, g), suggesting that the ATF4/DDIT4 axis may contribute to VCP inhibition-mediated paraptosis by positively affecting Akt activation and translational recovery. In addition, knockdown of Rictor but not Raptor potently inhibited Eer1-induced Akt activation, translational recovery, and ATF4/CHOP upregulation (Fig. 7h). Similar results were obtained by PP242 or LY294002 pretreatment (Fig. 7i). These results indicate the importance of the ATF4/DDIT4 axis and mTORC2/Akt signal in VCP inhibition-mediated paraptosis. Interestingly, mTORC2/Akt inhibition suppressed Eer1-induced ATF4 upregulation (Fig. 7h, i), and the knockdown of ATF4 or DDIT4 inhibited Eer1-induced Akt activation (Fig. 6n, o). Cross-regulation between the ATF4/DDIT4 axis and mTORC2/Akt signaling upon VCP inhibition suggests their cooperative role in translational recovery and proteotoxic stress enhancement.

### eIF3d may critically contribute to translational recovery in VCP inhibition-mediated paraptosis

**Fig. 8:**
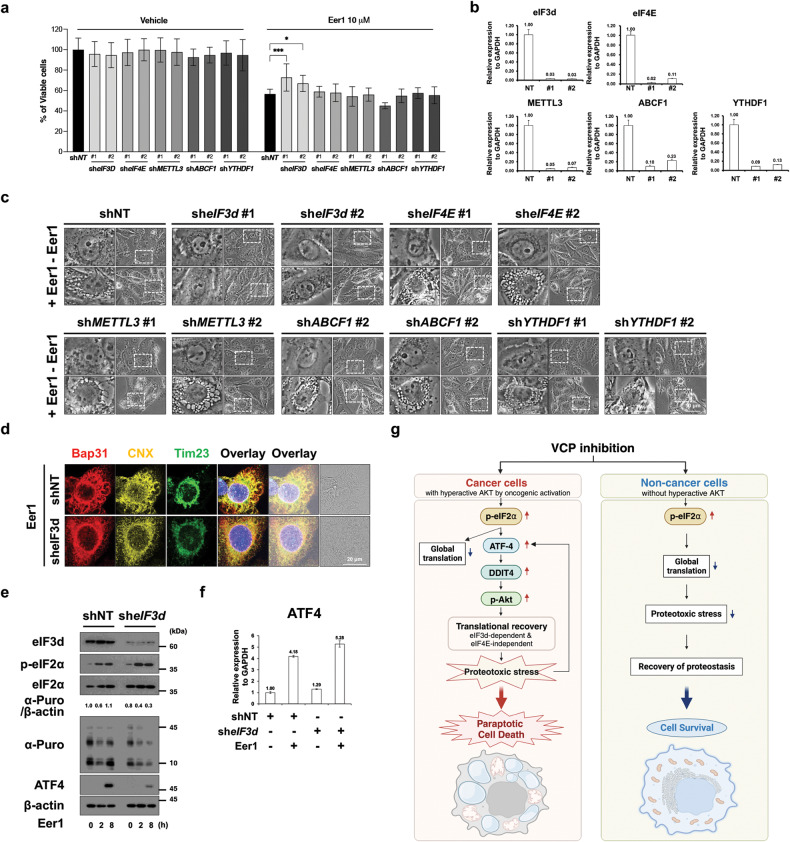
eIF3d critically contributes to VCP inhibition-mediated translational recovery and subsequent paraptosis. **a**–**f** MDA-MB 435 S cells transduced with the lentivirus encoding shRNAs against the indicated genes were treated with 10 μM Eer1 for 24 h **a**, 12 h **b**–**d**, indicated time duration **e** or 8 h **f**. **a** Cell viability. Data are presented as the means ± SD of three independent experiments. *n* = 10. The *p*-value was calculated by two-way ANOVA. *p < 0.05, ***p < 0.001. **b, f** qRT-PCR of the indicated genes using GAPDH as a reference gene. **c** Phase-contrast microscopy. **d** Immunocytochemistry. **e** Western blotting. **g** Hypothetical model for the cause of the preferential cytotoxicity of VCP inhibition to cancer cells with hyperactive Akt. While normal cells with low Akt activity are less sensitive to VCP inhibition due to translational suppression, cancer cells with hyperactive Akt due to oncogenic activation are more vulnerable to VCP inhibition-mediated paraptosis via enhanced proteotoxic stress triggered by ATF4/DDIT4/p-Akt-and eIF3d-mediated translational recovery. Therefore, cellular fates in response to VCP inhibition may depend on their Akt activity.

The mechanism underlying translation recovery in VCP inhibition-mediated paraptosis was further explored. It is known that under stress conditions, mTORC1-dependent cap-dependent mRNA translation is suppressed [41]. However, alternative mechanisms have been proposed to allow protein synthesis to adapt to various stressors. These mechanisms include eukaryotic translation initiation factor 3 subunit D (eIF3d), a subunit of the eIF3 complex with cap-binding activity) [42–45], as well as the m^6^A-pathway, which involves methyltransferase-like 3 (METTL3) [46], ATP Binding Cassette Subfamily F Member 1 (ABCF1) [47], and YTH N6-Methyladenosine RNA Binding Protein F1 (YTHDF1)) [48]. Remarkably, our findings revealed that eIF3d knockdown had a significant impact on Eer1-induced paraptosis (Fig. 8a–d), effectively inhibiting translational recovery (Fig. 8e). Intriguingly, this effect was not observed with knockdown of eIF4E, METTL3, ABCF1, or YTHDF1 (Fig. 8a–d). Furthermore, eIF3d knockdown resulted in the enhancement of eIF2α phosphorylation (Fig. 8e). Notably, eIF3d knockdown also suppressed the upregulation of ATF4 at the protein level without downregulating ATF4 mRNA levels (Fig. 8e, f). These findings underscore the pivotal role of eIF3d in facilitating translational recovery during VCP inhibition-induced paraptosis.

In summary, the selective cytotoxicity of VCP inhibition towards cancer cells can be attributed to the disruption of proteotoxic stress mitigation pathways involving the ATF4/DDIT4 axis and hyperactive mTORC2/Akt signaling. This process is further modulated by eIF3d-mediated translational recovery, which enhances proteotoxicity selectively in cancer cells undergoing paraptosis upon VCP inhibition (Fig. 8g).

## Discussion

Identifying cancer-selective targets and understanding their underlying mechanisms are pivotal in developing effective cancer therapies. Among these potential targets, VCP has emerged as both a prognostic biomarker and a prospective therapeutic target in cancer [8, 9]. Our study introduces a novel perspective by highlighting VCP’s central role as a molecular target in paraptosis, a distinctive form of programmed cell death. Importantly, our findings demonstrate that inhibiting VCP leads to preferential cell death in breast cancer cells compared to non-transformed cells, primarily through the induction of paraptosis. Genetic and pharmacological intervention of VCP commonly elicits the morphological features of paraptosis, reduction in cell viability, and ISR, demonstrating the crucial role of ATF4 in paraptosis. These results suggest that the gene-level intervention of VCP has the same mechanism of regulating paraptosis as that of VCP inhibitors.

The impairment of VCP-mediated ERAD and MAD processes appears central to this phenomenon. Our experiments revealed that Eer1, a VCP inhibitor, led to increased protein levels of the ERAD substrates (e.g., nuclear respiratory factor 1 (Nrf1) [49] and receptor accessory protein 5 (REEP5)) [50] and the MAD substrates (e.g., myeloid leukemia 1 (Mcl-1) [51] and mitofusin 2 (Mfn2) [52]) (see Supplementary Fig. 5). Inhibition of VCP may result in the progressive accumulation of misfolded proteins within the ER and mitochondria, leading to osmotic pressure changes and subsequent organelle swelling [53]. The fusion of the ER compartments induced by VCP inhibition may disrupt protein synthesis, folding, and transport, further exacerbating proteotoxic stress. Additionally, mitochondrial swelling and fusion at the early phase may act as an adaptive response to maintain mitochondrial membrane integrity [17]. However, excessive megamitochondrial expansion can compromise membrane potential, deplete cellular energy, and ultimately drive paraptotic cell death [17, 19]. Targeting these two organelles during paraptosis represents a unique and promising therapeutic strategy against solid tumors [19].

Proteasomal and VCP inhibitors both induce proteostatic stress [8, 54]. While proteasome inhibitors (PIs) have shown clinical utility in hematological malignancies by inducing apoptosis [55], their effectiveness against solid tumors has been limited [54, 56]. In contrast, various VCP inhibitors have demonstrated potent anti-tumor activities across various hematologic and solid tumor models [57]. The proteasome may not efficiently process ubiquitinated substrates, including those associated with ERAD, MAD, and chromatin-associated degradation, without the assistance of VCP [58–61]. Therefore, the preferential targeting of solid tumors by VCP inhibitors, compared to PIs, may be attributed to the broader defects in the UPS than those by PIs [57]. Additionally, compared to PIs, VCP inhibitors impact multiple cellular processes, including autophagy [62], endosomal trafficking [62, 63], DNA repair and genome stability [64], membrane fusion [65], non-proteolytic disassembly of protein phosphatase-1 complex [66, 67], and regulation of PD-L1 expression [68], possibly contributing to their efficacy in solid tumor models [31]. Among the developed VCP inhibitors, ATPase competitive inhibitors, CB-5083 and CB-5339, have reached clinical trials (https://clinicaltrials.gov trial number NCT02243917 & NCT04372641) by demonstrating effective anti-tumor activity across various tumor models [31, 69, 70]. Understanding the resistance mechanisms is crucial for developing more effective inhibitors or combination therapies. Resistance to VCP inhibitors, primarily attributed to specific mutations in the D2 ring ATPase domain and the linker region connecting the D1 and the D2 domains of VCP, presents a clinical challenge [57].

Recent findings from our laboratory have revealed distinct responses to PI treatment in different cell types [27]. Multiple myeloma (MM) cells were highly susceptible to bortezomib (Bz), inducing apoptosis, while breast cancer cells exhibited resistance. Interestingly, the application of ISRIB, a small molecule known to restore eIF2B-mediated translation during the integrated stress response, protected MM cells from apoptosis while enhancing Bz-mediated cytotoxicity in breast cancer cells by inducing paraptosis. These results suggest that enhancing translation and inducing paraptosis may effectively overcome PI resistance in solid tumor cells.

The present study further underscores that the difference in proteotoxic stress responses between cancer and normal cells could be exploited for therapeutic purposes. Sustained translation attenuation under VCP inhibition can alleviate proteotoxic stress and support the survival of non-transformed cells. However, translation recovery following initial suppression in cancer cells enhances proteotoxic stress, ultimately leading to paraptotic cell death.

The PI3K/Akt/mTOR signaling cascade is hyperactivated in many solid tumors, including breast cancer, contributing to cancer progression and resistance to pro-apoptotic therapies [71, 72]. However, targeting this pathway has shown limited efficacy due to feedback regulation and interference with other signaling pathways [73]. Our study reveals that VCP inhibition leads to selective mTORC2 activation and mTORC1 inhibition in cancer cells. In contrast, non-transformed cells exhibit mTORC1 activation without mTORC2 induction upon VCP inhibition. Additionally, our findings demonstrated that mTORC2 activation is essential for the selective action of VCP inhibitors in cancer cells. Inhibition of mTORC2/Akt signals effectively attenuates translational recovery and paraptosis induced by VCP inhibition. Cancer cells with hyperactive mTORC2/Akt signaling are more vulnerable to VCP inhibition, making VCP an attractive target in this context.

The ATF4/DDIT4/mTORC2/Akt signals are known to be required for cell survival under energy-related stresses, such as amino acid deprivation [39]. In our study, the ATF4/DDIT4 axis contributed to Akt activation, translational recovery, and paraptosis upon VCP inhibition. Additionally, eIF3d critically contributed to translational recovery, leading to ATF4 upregulation and enhancing cancer cells’ sensitivity. Therefore, we speculate that in response to proteotoxic stress, such as VCP inhibition, the ATF4/DDIT4/mTORC2/Akt signals and eIF3d may shift the cell fate towards paraptotic cell death.

In conclusion, our study unveils the potential of VCP as a therapeutic target in cancer, emphasizing the selective vulnerability of cancer cells to VCP inhibition-induced paraptosis. This strategy holds promise for overcoming resistance to pro-apoptotic therapies in solid tumors driven by oncogenic PI3K/Akt/mTOR signaling.

## Materials and methods

### Chemicals and antibodies

Chemicals were purchased from various sources: eeyarestatin-1 (Eer1), LY294002, PD98059, U0126, SP600125, and SB203580 from Calbiochem (EDM Millipore Corp., Billerica, MA, USA); CB-5083 from Biovision (Milpitas, California, USA); NMS-873 from APExBIO (Houston, TX 77014, USA); z-VAD-fmk from R&D Systems (Minneapolis, MN, USA); Necrostatin-1 (Nec-1), 3-methyladenine (3-MA), bafilomycin A1 (Bafilo), chloroquine (CQ), ferrostatin-1 (Ferro), and cycloheximide (CHX) from Sigma-Aldrich (St. Louis, MO, USA); PP242 and Torin1 from Selleckchem (Houston, TX 77014, USA); TRAIL from KOMA BIOTECH (Seoul, South Korea); MitoTracker-Red (MTR), tetramethylrhodamine methyl ester (TMRM), 4′,6-diamidino-2-phenylindole (DAPI), and propidium iodide (PI) from Molecular Probes (Eugene, OR, USA). The following antibodies were employed: VCP (#2648), GFP (#2555), p-eIF2α (#9721), eIF2α (#9722), CHOP (#2895), Nrf1(#8052), p-ERK1/2 (#9101), ERK (#9102), p-Akt (S473) (#9271), Akt (#9272), p-Akt (T308) (#9275), p-p70S6K (#9234), p70S6K (#2708), p-4EBP1 (#9451), 4EBP1 (#9452), Raptor (#2280), Rictor (#2114), and ATF4 (#11815) from Cell Signaling Technology (Danvers, MA, USA); β-actin (sc-47778), cytochrome C (sc-13156), Tom20 (sc-11415), ubiquitin (sc-8017), ATF4 (sc-200), and Mcl-1 (sc-819) from Santa Cruz (Dallas, TX, USA); α-Puromycin (MABE343) from Millipore (Billerica, MA, USA); Calnexin (CNX; PA5-19169) from Invitrogen (Carlsbad, CA, USA); Tim 23 (611222) from BD biotechnology (San Jose, CA, USA); Caspase-3 (ADI-AAP-113) from Enzo Life Sciences (Farmingdale, NY, USA); poly (ADP-ribose) polymerase (PARP; ab32071) and Bap31 (ab37120) from Abcam (Cambridge, UK); Ras (clone RAS10, #05-516) from Millipore; The secondary antibodies used were anti-rabbit IgG HRP (G-21234) and anti-mouse IgG HRP (G-21040) from Molecular Probes, Inc. (Eugene, OR, USA), and anti-rat IgG HRP from Sigma (A9037-1).

### Cell culture

Human breast cancer cell lines, the MCF10A human mammary epithelial cell line, and HEK-293T cells were acquired from the American Type Culture Collection (ATCC, Manassas, VA, USA). All cell lines underwent regular mycoplasma contamination checks, and their authenticity was confirmed through standard morphological examination using a microscope. The cell cultures were as follows: MDA-MB 231 and BT549 cells in RPMI-1640 medium (GIBCO-BRL, Grand Island, NY, USA); T47D and MDA-MB 468 cells in DMEM with high glucose (Hyclone, Logan, UT, USA); MDA-MB 435 S cells in DMEM with low glucose (Hyclone); Hs578T cells in DMEM high-glucose medium supplemented with 10 μg/ml insulin (Sigma-Aldrich, St. Louis, MO, USA); and MCF10A cells in DMEM/F12 medium supplemented with 5% horse serum, insulin, human epidermal growth factor, hydrocortisone, and cholera toxin (Calbiochem).

### Cell viability assay

All experiments were conducted in a low-glucose DMEM medium to exclude the effects of high glucose concentrations. Cells were cultured in 24-well plates (4×10^4^ cells per well), treated as indicated, fixed with methanol/acetone (1:1) at −20 °C for 5 min, washed with PBS, and stained with 1 μg/ml propidium iodide at room temperature for 10 min. Plates were imaged using an IncuCyte device (Essen Bioscience, Ann Arbor, MI, USA) and analyzed with IncuCyte ZOOM 2016B software. The IncuCyte program’s processing definition was set to identify attached (live) cells by their red-stained nuclei. The percentage of live cells was normalized to that of untreated control cells (100%).

### Immunoblot analysis

Immunoblot analysis was performed as described previously [33]. Representative results from at least three independent experiments are displayed, and unprocessed scans of immunoblots are provided as Source Data.

### Immunofluorescence microscopy

Following treatments, cells were fixed with acetone/methanol (1:1) for 5 min at −20 °C or with 4% paraformaldehyde for 10 min at room temperature. Fixed cells were blocked in 5% BSA in PBS for 30 min and incubated overnight at 4 °C with primary antibodies [BAP31 (rabbit, ab37120 from Abcam), Tim23 (mouse, 611222 from BD), CNX (goat, PA5-19169 from Invitrogen), cytochrome *c* (mouse, sc-13156 from Santa Cruz), or Tom20 (mouse, sc-17764 from Santa Cruz)] diluted (1:500) in blocking buffer. Cells were then washed and incubated with diluted (1:1000) anti-mouse or anti-rabbit Alexa Fluor 488 or 594 (Molecular Probes) for 1 h at room temperature. After mounting on slides with ProLong Gold antifade mounting reagent (Molecular Probes), cells were observed with a K1-Fluo confocal laser scanning microscope (Nanoscope Systems, Daejeon, Korea) using an appropriate filter set (excitation bandpass, 488 nm; emission bandpass, 525/50).

### Transmission electron microscopy

Cells were pre-fixed in Karnovsky’s solution (1% paraformaldehyde, 2% glutaraldehyde, 2 mM calcium chloride, 0.1 M cacodylate buffer, pH 7.4) for 2 h, post-fixed in 1% osmium tetroxide and 1.5% potassium ferrocyanide for 1 h, dehydrated with 50–100% alcohol, embedded in Poly/Bed 812 resin (Pelco, Redding, CA, USA), polymerized, and observed under an electron microscope (EM 902 A, Carl Zeiss, Oberkochen, Germany).

### Mouse xenograft studies

Animal experiments adhered to the guidelines and regulations approved by the Institutional Animal Care and Use Committees of Asan Institute for Life Science (approval number 2017-12-091, granted on May 02, 2017). Female BALB/c nude mice (nu/nu, 5 weeks old; Japan SLC, Hamamatsu, Japan) were injected in the right flank with MDA-MB 435 S cells (5 × 10^6^ cells/mouse). Tumors were allowed to grow for 3 weeks until the average tumor volume reached 100–150 mm^3^. Mice were randomized into three groups (*n* = 5 per group) and received oral administration (O.A.; qd4/3off) of vehicle (PBS containing 0.25% DMSO), 100 mg/kg CB-5083, or 150 mg/kg CB-5083. Researchers were blinded to the group allocations during the experiment and when assessing the outcome. Tumor size was measured twice a week for 2 weeks, and tumor volume was calculated. On the 15^th^ day, mice were sacrificed, and the tumors were isolated, fixed in 4% paraformaldehyde, and embedded in paraffin. Tissue sections stained with H&E were observed under a K1-Fluo microscope (Nanoscope Systems) and photographed using a complementary metal-oxide-semiconductor (CMOS) camera.

### Construction of plasmids encoding mCherry-VCP WT and mCherry-VCP QQ

mCherry-VCP WT and mCherry-VCP QQ were generated from the plasmids VCP (wt)-AdditionEGFP (#23971) and VCP(DK0)-EGFP (VCP QQ) (#23974) (Addgene, Watertown, MA, USA), respectively, using the pENTRY/pDEST-mCherry system (Invitrogen). The fragments encoding VCP WT and VCP QQ were PCR amplified using the following primers: forward (ATGGCTTCTGGAGCCGATTCA) and reverse (GCCATACAGGTCATCVATCATT). These fragments were used to generate the pENTRY-VCP WT and pENTRY-VCP QQ vectors. Subsequently, mCherry-VCP WT and mCherry-VCP QQ were generated by recombining the pENTRY-VCP WT or pENTRY-VCP QQ vector with a pCS-mCherry vector utilizing the Gateway LR cloning system from Invitrogen.

### Generation and preparation of recombinant adenoviruses expressing VCP WT-EGFP and VCP QQ-EGFP

Replication-incompetent adenovirus expressing VCP WT-EGFP or VCP QQ-EGFP were generated as described previously [74, 75]. The DNA fragment encoding the VCP WT-EGFP- or VCP QQ-EGFP was excised from the respective plasmids (VCP WT-EGFP (#23971) and VCP(DKO)-EGFP (#23974), Addgene) using *BamH*1 and *Bgl*II restriction enzymes. These fragments were then ligated with the *BamH*1-digested adenoviral shuttle vector, pCA14. The resulting constructs, pCA14/VCP WT-EGFP and pCA14/VCP QQ-EGFP, were linearized by *Pvu*I digestion. The E1/E3-deleted adenoviral vector, dE1-RGD, was also linearized by *BstB*I digestion. These linearized vectors were co-transformed into *E. coli* BJ5183 competent cells for homologous recombination. The resulting adenoviral plasmids, dE1/VCP WT-EGFP and dE1/VCP QQ-EGFP, were digested with *Pac*I and transfected into 293 A cells. Finally, adenoviruses expressing VCP WT-EGFP or VCP QQ-EGFP were propagated, amplified in 293 A cells, and purified using cesium chloride density gradient centrifugation.

### Small interfering RNA-mediated gene silencing

siRNA Negative Control (siNC) (Stealth RNAi^TM^, 12935300) was purchased from Invitrogen (Carlsbad, CA, USA). VCP-targeted siRNAs were acquired from QIAGEN (Hilden Düsseldorf, NRW, Germany). These included siVCP #1 (target sequence AACAGCCATTCTCAAACAGAA), siVCP #2 (target sequence ATCCGTCGAGATCACTTTGAA), and siVCP #3 (target sequence AAGATGGATCTCATTGACCTA). CHOP (*DDIT3*) targeted siRNA (target sequence GAGCUCUGAUUGACCGAAUGGUGAA) was synthesized by Invitrogen. siATF4 (target sequences: CCACUCCAGAUCAUUCCUU, GGAUAUCACUGAAGGAGAU, and GUGAGAAACUGGAUAAGAA, sc-35112) was obtained from Santa Cruz. The siRNA oligonucleotides were annealed and transfected into cells using the RNAiMAX reagent (Invitrogen) following the manufacturer’s instructions. Western blotting was performed to confirm successful siRNA-mediated knockdown.

### Lentivirus-mediated shRNA transduction

To generate the lentiviral vectors encoding short hairpin RNA (shRNA), the pLKO.1 neo plasmid (#13425: Addgene, Cambridge, MA, USA) was digested using *Age*I and *EcoR*I. Two oligonucleotide strands were mixed and incubated at 95 °C for 4 min, and then at 70 °C for 10 min before slowly cooling to room temperature. The annealed oligo pair was ligated into the digested pLKO.1 neo plasmid using T4 ligase at 20 °C for 16 h. The sequences of the oligonucleotides used to knock down each target gene are listed in Supplementary Table 1. To produce the lentivirus containing each plasmid, HEK-293T cells were transfected with the lentiviral vector in the presence of pMD2.G/psPAX2.0 using linear polyethyleneimine (MW2,500; Polysciences, Warrington, PA, USA). Following transfection, the virus-containing supernatants were filtered, combined with polybrene, and used to infect MDA-MB 435 S cells. qRT-PCR and Western blot analyses were performed to validate the efficiency of transfection. The sequences of the shRNA are provided in Supplementary Table 1.

### Quantitative Real-Time RT-PCR (qRT-PCR)

Total RNA was extracted using the TRIzol® reagent (Invitrogen). Subsequently, cDNA was synthesized using 1 μg of total RNA with the M-MLV cDNA Synthesis kit (EZ006S; Enzynomics, Daejeon, Korea). Quantitative real-time polymerase chain reaction (qRT-PCR) was conducted using a Bio-Rad Real-Time PCR System (Bio-Rad, Richmond, CA, USA). The results were analyzed using the 2^–ΔΔCt^ method [76]. Primers for qRT-PCR are listed in Supplementary Table 2.

### Establishment of MCF10A cell lines stably expressing HRas^G12V^ and KRas^G12V^

To establish cell lines expressing *HRas*^*G12V*^ and *KRas*^*G12V*^, GP2-293 packaging cells were co-transfected with pVSV-G (#631530: Clontech, Mountain View, CA, USA) along with either pBABE-puro, pBABE puro H-Ras V12, or pBABE puro K-Ras V12 (#9051, #9052, or #1764: Addgene) using a CalPhos™ Mammalian Transfection Kit (#631312, Clontech) following the manufacturer’s instructions. Retroviral supernatants were used to transduce MCF10A cells in the presence of polybrene (5 mg/mL; Millipore, Burlington, MA, USA). Transduced cells were selected with puromycin (Invivogen, San Diego, CA, USA) for 3 weeks. Selected single cells were isolated, and the expression of HRas^G12V^ and KRas^G12V^ was confirmed by Western blotting.

### Morphological examination of ER and mitochondria

Cell lines stably expressing fluorescence in the ER lumen (YFP-ER cells), ER membrane (Sec61β-GFP cells), or mitochondria (YFP-Mito cells) [16] were used for morphological studies. YFP-ER cells were stained with 100 nM MitoTracker-Red (MTR) for 10 min to observe both the ER and mitochondria. Confocal microscopy was performed using a K1-Fluo confocal laser scanning microscope (Nanoscope Systems, Daejeon, Korea) with an appropriate filter set (excitation bandpass, 488 nm; emission bandpass, 525/50).

### Analysis of protein synthesis by puromycin labeling

Protein synthesis was monitored using the SUnSET method [40]. Briefly, newly synthesized peptides in cultured cells were labeled by adding 10 μg/ml puromycin for 10 min before cell collection. Whole-cell extracts were prepared for Western blotting using an anti-puromycin antibody (Millipore) and anti-mouse IgG-HRP-linked antibody (Molecular Probes). Fold changes in the protein levels of interest compared to β-actin were calculated following densitometric analysis.

### Statistical analysis

All experiments were repeated at least three times. Data were presented as mean ± standard deviation. Statistical analysis was performed using GraphPad Prism 9 (Graph Pad Software Inc, San Diego, CA, USA). The normality of data was assessed using Kolmogorov–Smirnov tests, and equal variance was assessed using Bartlett’s test. For normally distributed data, statistical differences were determined using analysis of variance (ANOVA), followed by the Bonferroni multiple comparison test. For all tests, *p* < 0.05 was considered significant (ns not significant, *p < 0.05, **p < 0.01, ***p < 0.001, **** *p* < 0.0001).

### Supplementary information

Supplementary Information

Uncropped Western Blot

## Data Availability

All data and information concerning this study will be provided upon request.
