# Supplementary material for: Akt enhances the vulnerability of cancer cells to VCP/p97 inhibition-mediated paraptosis
Source: Cell Death Dis. 2024 Jan 13;15(1):48. doi: 10.1038/s41419-024-06434-x (PMC10787777; doi:10.1038/s41419-024-06434-x)
Supplement: Supplementary file 1 — Supplementary Information [file 41419_2024_6434_MOESM1_ESM.docx]

**Supplementary Information**

**Akt enhances the vulnerability of cancer cells to VCP/p97 inhibition-mediated paraptosis**

**Dong Min Lee^1,2,#^, In Young Kim^1,2,#^, Hong Jae Lee^1,2^, Min Ji Seo^1,2^, Mi-Young Cho^1,2^, Hae In Lee^1,2^, Gyesoon Yoon^1,2^, Jae-Hoon Ji^3,4^, Seok Soon Park^5^, Seong-Yun Jeong^5^, Eun Kyung Choi^5^, Yong Hyeon Choi^6^, Chae-Ok Yun^6^, Mirae Yeo^7^, Eunhee Kim^7,*^, Kyeong Sook Choi^1,2*^**

^1^Department of Biochemistry and Molecular Biology, Ajou University School of Medicine, Suwon 16499, Republic of Korea

^2^Department of Biomedical Sciences, Ajou University Graduate School of Medicine, Suwon 16499, Republic of Korea

^3^Department of Biochemistry and Structural Biology, University of Texas Health at San Antonio, San Antonio, Texas 78229, USA

^4^Greehey Children's Cancer Research Institute, University of Texas Health at San Antonio, San Antonio, Texas 78229, USA

^5^Asan Institute for Life Sciences, Department of Convergence Medicine, Asan Medical Center, University of Ulsan College of Medicine, Seoul, 05505, Korea

^6^Department of Bioengineering, College of Engineering, Hanyang University, Seoul 04763, Korea

^7^Department of Biological Sciences, Ulsan National Institute Science and Technology, Ulsan 44919, South Korea

*Corresponding Author: Eunhee Kim, Ph.D. E-mail: ehkim@unist.ac.kr

Kyeong Sook Choi, Ph.D. E-mail: kschoi@ajou.ac.kr

# Both authors equally contributed to this work.

**
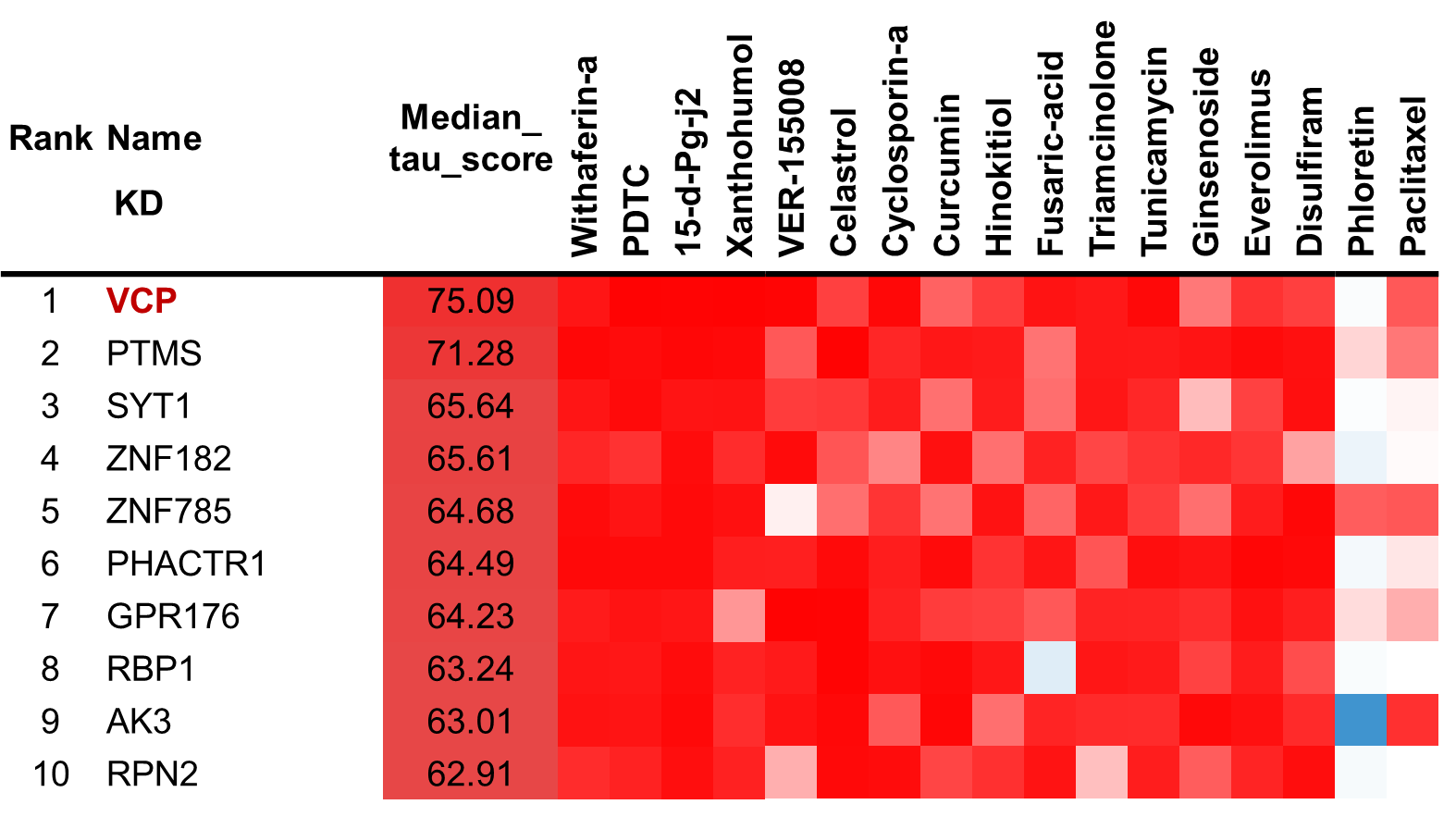
**

**Supplementary Fig. 1. Identification of VCP as a potential target gene of paraptosis through CMAP analysis.**

In the pursuit of potential target genes linked to paraptosis, we utilized the Connectivity Map (CMAP) CLUE web application (<http://cleu.io>) resource. Our approach involved cross-referencing the expression signatures of 17 paraptosis-inducing chemicals with the CMAP database. We aimed to pinpoint genes whose knockdown resulted in transcriptomic profiles resembling those induced by these chemicals. From this analysis, we identified 10 candidate genes based on their connectivity scores, with VCP emerging as one of the prominent candidates in this analysis.

**
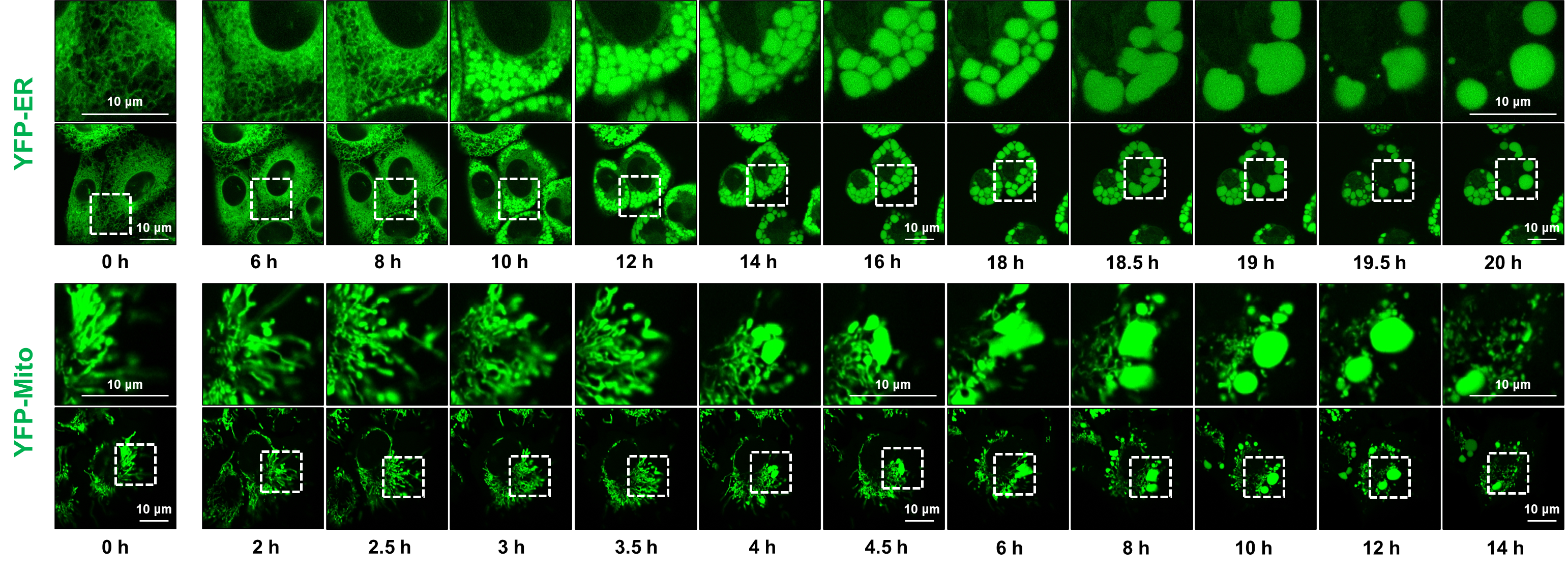
**

**Supplementary Fig. 2.** Live-cell time-lapse imaging in Eer1-treated YFP-ER cells and YFP-Mito cells.

YFP-ER or YFP-Mito cells were seeded in a 12-well plate (BD Biosciences) and subjected to treatment with 10 μM Eer1 within a Stage-top Incubator System TC (Live Cell Instrument, Seoul, Korea). A K1-Fluo confocal microscope system (Nanoscope Systems) was employed to capture images at 30-min intervals for 24-h period.

**
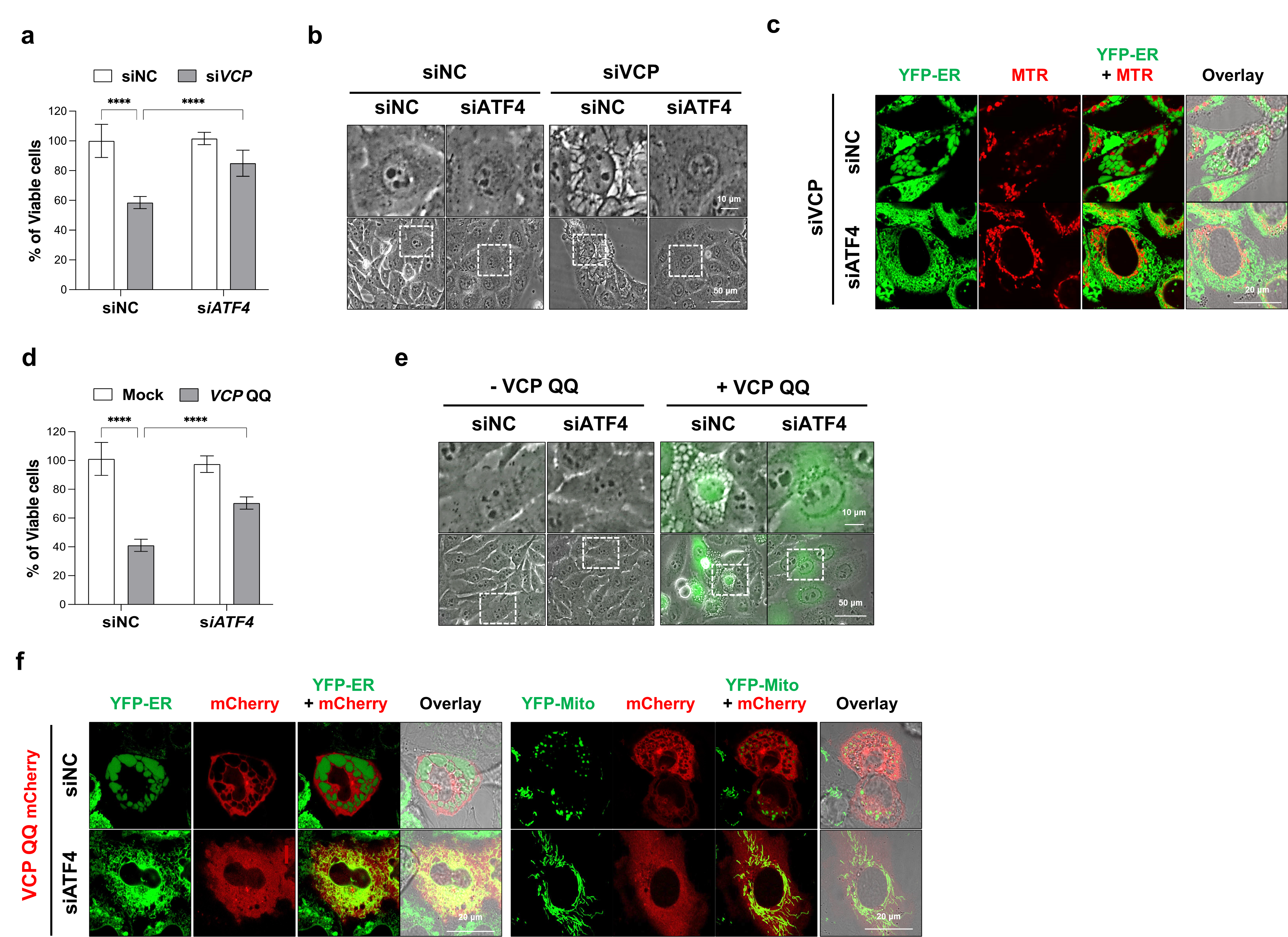
**

**Supplementary Figure 3. ATF4 knockdown attenuates paraptosis induced by VCP knockdown or VCP dominant mutant.**

**a, b** MDA-MB 435S cells transfected with siNT or siATF4 were further transfected with siNC or siVCP for 16 h (**a**) or 8 h (**b**). **a** Cell viability assay. **b** Phase-contrast microscopy. **c** Confocal microscopy in YFP-ER cells transfected with siNC or siATF4, followed by transfection with siVCP for 16 h, and stained with MTR. **d, e** MDA-MB 435S cells infected with adenovirus encoding VCP QQ-EGFP were further transfected with siNC or siATF4 for 48 h (**d**) or 36 h (**e**). **d** Cell viability assay. **e** Fluorescence/phase-contrast microscopy. **f** Confocal microscopy in YFP-ER or YFP-Mito cells transfected with siNC or siATF4, further infected with adenovirus encoding VCP QQ mCherry for 36 h.

**
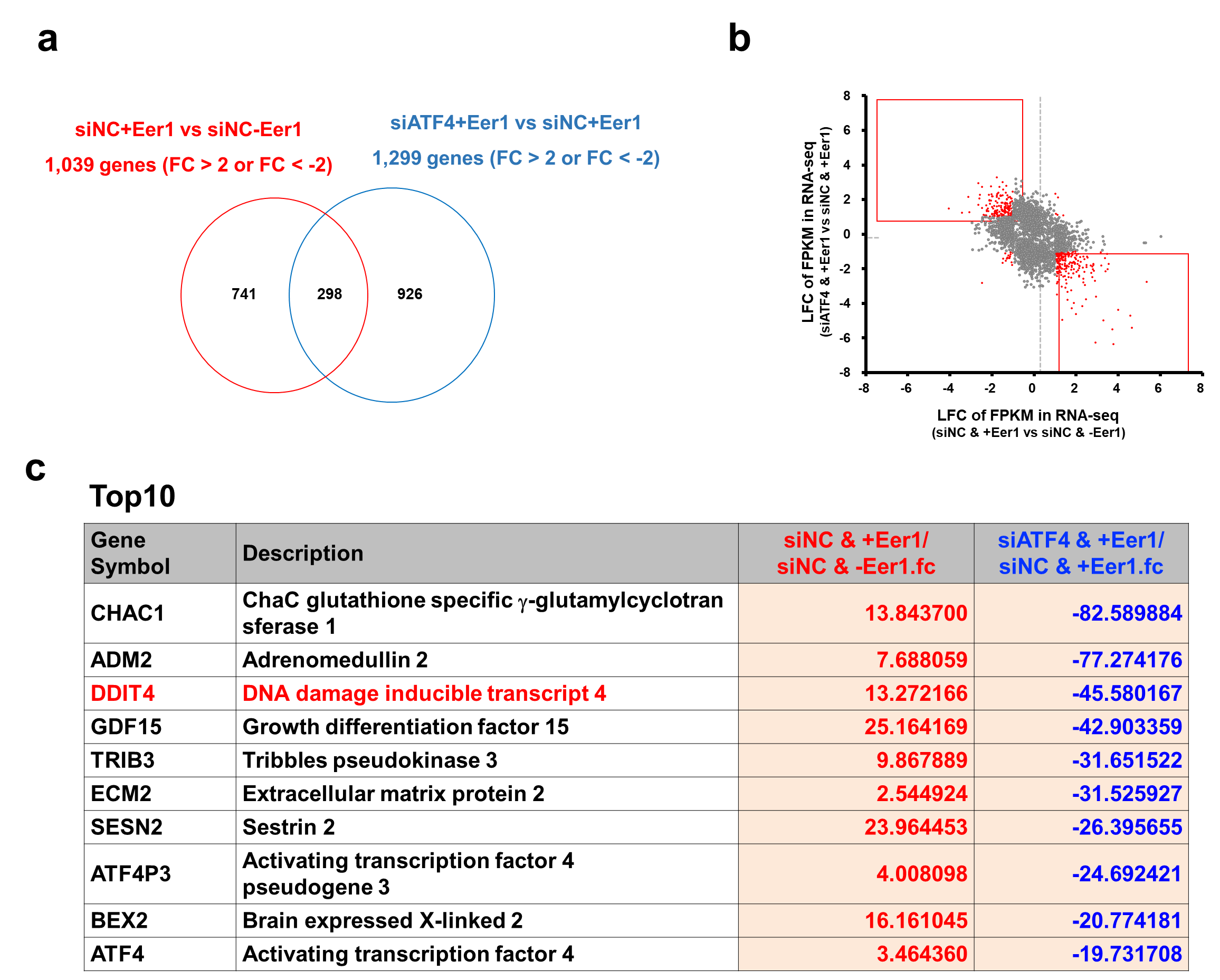
**

**Supplementary Fig. 4. Transcriptome analysis in MDA-MB 435S cells after transfection with or without siATF4 and treatment with or without Eer1.**

An RNA sequencing and transcriptome analysis were conducted on MDA-MB 435S cells subjected to four different conditions: siNC-transfected and untreated (siNC-Eer1), siNC-transfected and Eer1-treated (siNC+Eer1), siATF4-transfected and untreated (siATF4-Eer1), and siATF4-transfected and Eer1-treated (siATF4+Eer1). MDA-MB 435S cells were transfected with either siNC or siATF4 and subsequently treated with or without Eer1. Total RNA was extracted using the TRIzol® reagent (#10296010, Thermo Scientific, Waltham, MA, USA), and sequencing libraries were prepared using the TruSeq stranded messenger RNA library kit designed for the Illumina platform (San Diego, CA, USA). Following RNA sequencing, RNA-seq reads were aligned to the human reference genome (GRCh37) using HISAT2 version 2.1.0 and Bowtie2 2.3.4.1., and then assembled using StringTie version 2.1.3b. Differentially expressed genes (DEGs) were identified using DESeq2, with genes exhibiting a fold change ≥ 2 and an adjusted p-value < 0.05 considered as DEGs. **a** The Venn diagram illustrates the overlap of differentially expressed genes (DEGs) between the siNC+Eer1 group and the siATF4+Eer1 group. **b** A scatterplot representing the DEGs based on Eer1 treatment and ATF4 knockdown is presented. **c** The dependency of Eer1-responsive genes on ATF4 was assessed.

**
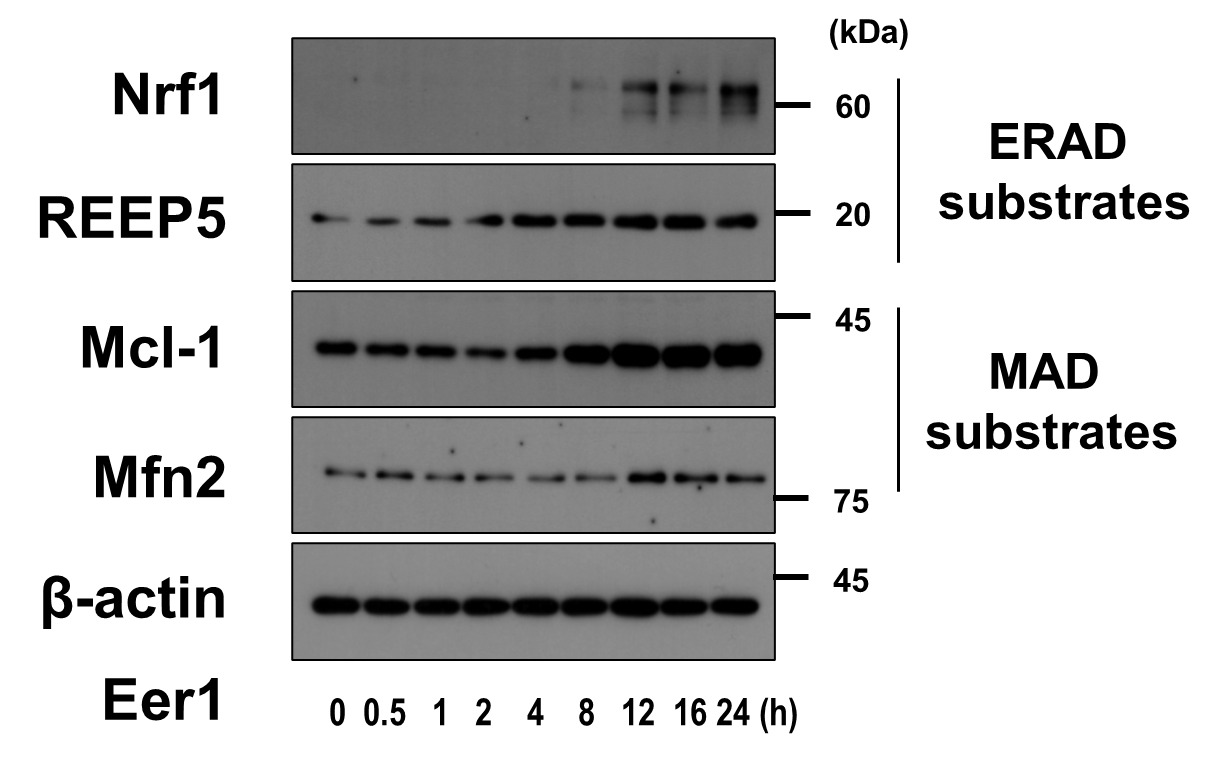
**

**Supplementary Figure 5. VCP inhibition leads to the accumulation of ERAD and MAD substrates.**

Western blotting was performed on MDA-MB 435S cells treated with 10 μM Eer1 for varying durations to examine the levels of the indicated proteins.

**Supplementary Table 1. The nucleotide sequences for shRNAs.**

| Name | | Sequences (5’ to 3’) |
| --- | --- | --- |
| Raptor | #1_Sense | CCGGGGCTAGTCTGTTTCGAAATTTCTTCCTGTCAAAATTTCGAAACAGACTAGCCTTTTTG |
|  | #1_AntiS | AATTCAAAAAGGCTAGTCTGTTTCGAAATTTTGACAGGAAGAAATTTCGAAACAGACTAGCC |
|  | #2_Sense | CCGGAGGGCCCTGCTACTCGCTTTTCTCGAGAAAAGCGAGTAGCAGGGCCCTTTTTTG |
|  | #2_AntiS | AATTCAAAAAAGGGCCCTGCTACTCGCTTTTCTCGAGAAAAGCGAGTAGCAGGGCCCT |
| Rictor | #1_Sense | CCGGTACTTGTGAAGAATCGTATCTTCTCGAGAAGATACGATTCTTCACAAGTTTTTTG |
|  | #1_AntiS | AATTCAAAAAATACTTGTGAAGAATCGTATCTTCTCGAGAAGATACGATTCTTCACAAG |
|  | #2_Sense | CCGGTACTTGTGAAGAATCGTATCTTCTCGAGAAGATACGATTCTTCACAAGTTTTTTG |
|  | #2_AntiS | AATTCAAAAAACTTGTGAAGAATCGTATCTTCTCGAGAAGATACGATTCTTCACAAGTA |
| eIF4E | #1_Sense | CCGGCGGCTGATCTCCAAGTTTGATCTCGAGATCAAACTTGGAGATCAGCCGTTTTTG |
|  | #1_AntiS | AATTCAAAAACGGCTGATCTCCAAGTTTGATCTCGAGATCAAACTTGGAGATCAGCCG |
|  | #2_Sense | CCGGCCACTCTGTAATAGTTCAGTACTCGAGTACTGAACTATTACAGAGTGGTTTTTG |
|  | #2_AntiS | AATTCAAAAACCACTCTGTAATAGTTCAGTACTCGAGTACTGAACTATTACAGAGTGG |
| METTL3 | #1_Sense | CCGGCGTCAGTATCTTGGGCAAGTTCTCGAGAACTTGCCCAAGATACTGACGTTTTTG |
|  | #1_AntiS | AATTCAAAAACGTCAGTATCTTGGGCAAGTTCTCGAGAACTTGCCCAAGATACTGACG |
|  | #2_Sense | CCGGGCCAAGGAACAATCCATTGTTCTCGAGAACAATGGATTGTTCCTTGGCTTTTTG |
|  | #2_AntiS | AATTCAAAAAGCCAAGGAACAATCCATTGTTCTCGAGAACAATGGATTGTTCCTTGGC |
| YTHDF1 | #1_Sense | CCGGGTTCGTTACATCAGAAGGATACTCGAGTATCCTTCTGATGTAACGAACTTTTTG |
|  | #1_AntiS | AATTCAAAAAGTTCGTTACATCAGAAGGATACTCGAGTATCCTTCTGATGTAACGAAC |
|  | #2_Sense | CCGGCCCTACCTGTCCAGCTATTACCTCGAGGTAATAGCTGGACAGGTAGGGTTTTTG |
|  | #2_AntiS | AATTCAAAAACCCTACCTGTCCAGCTATTACCTCGAGGTAATAGCTGGACAGGTAGGG |
| ABCF1 | #1_Sense | CCGGGCAGAGTGTTAGCCAAATCGACTCGAGTCGATTTGGCTAACACTCTGCTTTTTG |
|  | #1_AntiS | AATTCAAAAAGCAGAGTGTTAGCCAAATCGACTCGAGTCGATTTGGCTAACACTCTGC |
|  | #2_Sense | CCGGGCCAAGTTTATGTGGCCTATTCTCGAGAATAGGCCACATAAACTTGGCTTTTTTG |
|  | #2_AntiS | AATTCAAAAAAGCCAAGTTTATGTGGCCTATTCTCGAGAATAGGCCACATAAACTTGGC |
| eIF3d | #1_Sense | CCGGGACGACATGGATAAGAATGAACTCGAGTTCATTCTTATCCATGTCGTCTTTTTTG |
|  | #1_AntiS | AATTCAAAAAAGACGACATGGATAAGAATGAACTCGAGTTCATTCTTATCCATGTCGTC |
|  | #2_Sense | CCGGGCGTCATTGACATCTGCATGACTCGAGTCATGCAGATGTCAATGACGCTTTTTTG |
|  | #2_AntiS | AATTCAAAAAAGCGTCATTGACATCTGCATGACTCGAGTCATGCAGATGTCAATGACGC |
| DDIT4 | #1_Sense | CCGGACCGGCTTCAGAGTCATCAAGCTCGAGCTTGATGACTCTGAAGCCGGTTTTTTG |
|  | #1_AntiS | AATTCAAAAAAACCGGCTTCAGAGTCATCAAGCTCGAGCTTGATGACTCTGAAGCCGG |
|  | #2_Sense | CCGGGTTAAGTTCTGCCAACTCTTCCTCGAGGAAGAGTTGGCAGAACTTAACTTTTTTG |
|  | #2_AntiS | AATTCAAAAAAGTTAAGTTCTGCCAACTCTTCCTCGAGGAAGAGTTGGCAGAACTTAAC |

**Table 2. The primer sequences for qRT-PCR**

| Gene | Reverse primer sequence (5’3’) | |
| --- | --- | --- |
| Raptor | Forward | ATTCTCGCCGTGATCGTCAA |
|  | Reverse | GGAGAAGGCAAGGCGTAGTT |
| Rictor | Forward | AGAACCTCCGAGTACGAGGG |
|  | Reverse | GCCACCACCTCTGGATTCTG |
| eIF4E | Forward | CGGAATCTAATCAGGAGGTTGC |
|  | Reverse | GATCAGCCGCAGGTTTGC |
| METTL3 | Forward | AGCCTTCTGAACCAACAGTCC |
|  | Reverse | CCGACCTCGAGAGCGAAAT |
| YTHDF1 | Forward | GCACACAACCTCCATCTTCG |
|  | Reverse | AACTGGTTCGCCCTCATTGT |
| ABCF1 | Forward | AGAAAGCCCGAGTTGTGTTTG |
|  | Reverse | GCCCCCTTGTAGTCGTTGATG |
| eIF3d | Forward | CTGGAGGAGGGCAAATACCT |
|  | Reverse | CTCGGTGGAAGGACAAACTC |
| ATF4 | Forward | CTCCGGGACAGATTGGATGTT |
|  | Reverse | GGCTGCTTATTAGTCTCCTGGAC |
| CHOP | Forward | GGAAACAGAGTGGTCATTCCC |
|  | Reverse | CTGCTTGAGCCGTTCATTCTC |
| DDIT4 | Forward | TGAGGATGAACACTTGTGTGC |
|  | Reverse | CCAACTGGCTAGGCATCAGC |
| GAPDH | Forward | GAGTCAACGGATTTGGTCGT |
|  | Reverse | TGGAAGATGGTGATGGGATT |

qRT-PCR, quantitative real-time polymerase chain reaction; Raptor, regulatory-associated protein of mTOR; Rictor, RPTOR Independent Companion Of MTOR Complex 2; eIF4E, Eukaryotic Translation Initiation Factor 4E; METTL3, methyltransferase 3; YTHDF1, YTH N6-Methyladenosine RNA Binding Protein 1; ABCF1, ATP-binding cassette F1; eIF3d, Eukaryotic translation initiation factor 3 subunit D; ATF4, Activating Transcription Factor 4; CHOP, C/EBP-homologous protein; DDIT4, DNA Damage-Inducible Transcript 4; GAPDH, glyceraldehyde 3-phosphate dehydrogenase
