# Supplementary figures and images for: Akt enhances the vulnerability of cancer cells to VCP/p97 inhibition-mediated paraptosis
Source: Cell Death Dis. 2024 Jan 13;15(1):48. doi: 10.1038/s41419-024-06434-x (PMC10787777; doi:10.1038/s41419-024-06434-x)

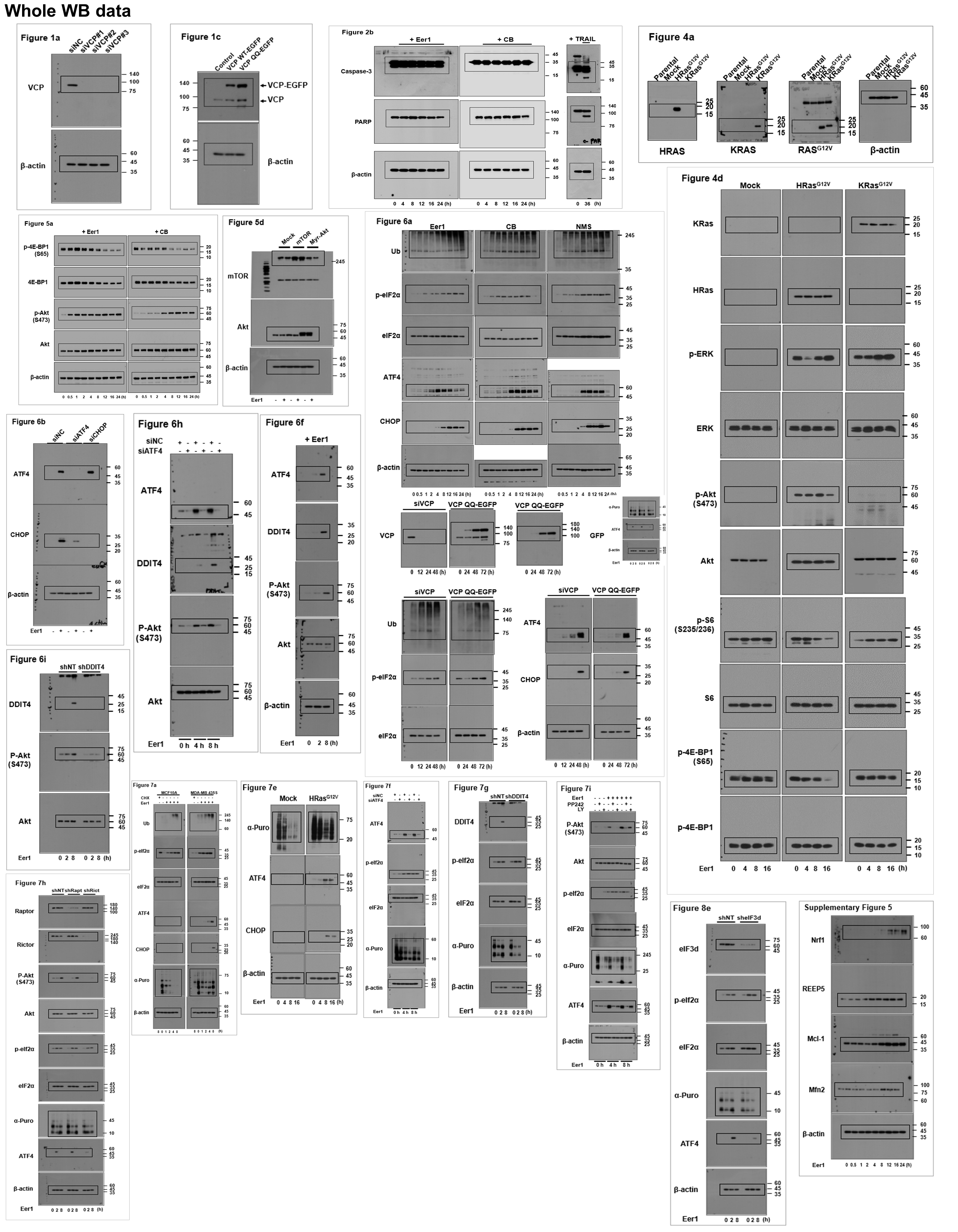

Supplement: Supplementary file 2 — Uncropped Western Blot [file 41419_2024_6434_MOESM2_ESM.tif]
